# A Mathematical Model of Lysosomal Ion Homeostasis Points to Differential Effects of Cl^−^ Transport in Ca^2+^ Dynamics

**DOI:** 10.3390/cells8101263

**Published:** 2019-10-16

**Authors:** Rosario Astaburuaga, Orlando Daniel Quintanar Haro, Tobias Stauber, Angela Relógio

**Affiliations:** 1 Institute for Theoretical Biology (ITB), Charité-Universitätsmedizin Berlin, Corporate Member of the Freie Universität Berlin, Humboldt-Universität zu Berlin, and Berlin Institute of Health, 10115 Berlin, Germany; rosario.astaburuaga@charite.de (R.A.); odqh@hotmail.com (O.D.Q.H.); 2 Medical Department of Hematology, Oncology and Tumor Immunology, Molekulares Krebsforschungzentrum (MKFZ), Charité-Universitätsmedizin Berlin, Corporate Member of the Freie Universität Berlin, Humboldt-Universität zu Berlin, and Berlin Institute of Health, 13353 Berlin, Germany; 3 Freie Universität Berlin, Institute of Chemistry and Biochemistry, 14195 Berlin, Germany; 4 Department of Human Medicine, Medical School Hamburg, 20457 Hamburg, Germany

**Keywords:** mathematical modelling, lysosomal homeostasis, slowly voltage-gated chloride transport, lysosomal Ca^2+^ dynamics

## Abstract

The establishment and maintenance of ion gradients between the interior of lysosomes and the cytosol are crucial for numerous cellular and organismal functions. Numerous ion transport proteins ensure the required variation in luminal concentrations of the different ions along the endocytic pathway to fit the needs of the organelles. Failures in keeping proper ion homeostasis have pathological consequences. Accordingly, several human diseases are caused by the dysfunction of ion transporters. These include osteopetrosis, caused by the dysfunction of Cl^−^/H^+^ exchange by the lysosomal transporter ClC-7. To better understand how chloride transport affects lysosomal ion homeostasis and how its disruption impinges on lysosomal function, we developed a mathematical model of lysosomal ion homeostasis including Ca^2+^ dynamics. The model recapitulates known biophysical properties of ClC-7 and enables the investigation of its differential activation kinetics on lysosomal ion homeostasis. We show that normal functioning of ClC-7 supports the acidification process, is associated with increased luminal concentrations of sodium, potassium, and chloride, and leads to a higher Ca^2+^ uptake and release. Our model highlights the role of ClC-7 in lysosomal acidification and shows the existence of differential Ca^2+^ dynamics upon perturbations of Cl^−^/H^+^ exchange and its activation kinetics, with possible pathological consequences.

## 1. Introduction

Lysosomes are membrane-enclosed organelles of eukaryotic cells characterized by an acidic pH, an enrichment in hydrolytic enzymes and a specific composition of membrane proteins [1,2]. These highly specialized organelles are the major cellular compartment for the degradation of proteins, carbohydrates, lipids and nucleic acids delivered by endocytosis and phagocytosis from the extracellular space, or by autophagy. During the last years, lysosomes have also been recognized as important platforms for nutrient sensing and metabolic signalling [3,4,5]. In addition, lysosomes and lysosome-related organelles are key players in various processes like plasma membrane repair, antigen presentation and bone resorption [6,7]. Lysosomal dysfunction causes rare lysosomal storage diseases [8,9] and is additionally associated with neurodegenerative disorders, such as Parkinson’s and Alzheimer’s diseases [10], and cancer [5,11,12]. The circadian clock was recently also shown to be influenced by the spatial distribution of lysosomes, which correlates with lysosomal homeostasis [13,14], via the mTORC1 pathway [15].

To fulfil their cell physiological functions, lysosomes require a particular luminal ion composition, which is established and maintained by a plethora of ion transport proteins, such as ion pumps, transporters and channels [16,17]. These include the energy-consuming V-ATPase, which actively pumps protons from the cytosol into the lysosome, generating the required acidic internal pH of about 4.5 [18,19,20]. Since the lysosomal lumen exhibits a considerable buffering capacity for protons (H^+^), 30–60 mM of H^+^ has to be pumped into the lumen in order to decrease the pH by one unit [21]. This electrogenic process requires a parallel electrical shunt by cation efflux and/or anion influx to prevent a rapid build-up of an inside-positive potential that would inhibit further acidification. In situ measurements of the lysosomal transmembrane potential showed inside-positive values of +20 mV [22] or even up to +100 mV [23], and recent electrophysiological studies on enlarged lysosomes with defined ionic solutions reported contradictory results, either inside-positive [24] or inside-negative potentials [25,26].

Calcium ions (Ca^2+^) have been shown to be of pivotal importance to lysosomal trafficking and function [27,28]. Lysosomal Ca^2+^ release is important for several cellular processes including lysosomal fusion and exocytosis. Various cues may trigger the opening of Ca^2+^ release channels, such as NAADP, the generation of PI(3,5)P_2_ or mTOR signalling. Lysosomes accumulate Ca^2+^ to a free concentration of about 0.5 mM, which is more than 5000-fold higher than the resting cytosolic [Ca^2+^] of approximately 100 nM [28,29,30]. Yet, the uptake mechanism and the protein(s) involved in lysosomal Ca^2+^ accumulation remain unknown. Its dependence on the acidic lysosomal pH suggested the existence of a direct or indirect H^+^/Ca^2+^ exchange [28,30,31]. Ca^2+^/proton exchangers of the CAX family have been shown to mediate vacuolar Ca^2+^ uptake in plants and fungi [28]. Recently orthologues of CAX were also shown to be present in animals, excluding placental mammalia [31]. However, this pH dependence of lysosomal Ca^2+^ accumulation has been questioned [26,32,33,34]. Several proteins have been identified that mediate Ca^2+^ release from lysosomes, including transient receptor potential cation channel mucolipin subfamily proteins (TRPMLs) and two-pore channels (TPCs) [4,34,35,36]. More recently, the voltage-gated CACNA1A [37] and the ligand-gated P2X4 Ca^2+^ channels were shown to localize on lysosomes [38].

The monovalent cations sodium (Na^+^) and potassium (K^+^) constitute the main positive charge of the lysosomal lumen. There are conflicting data regarding their luminal concentrations, with values for Na^+^ ranging from 20 to 140 mM, depending on the experimental setup [39,40]. While the transport of Na^+^ is often coupled to that of metabolites and can additionally be mediated by the Na^+^-conductance of TPCs [40,41], several K^+^-specific channels have recently been identified on lysosomes. These comprise TMEM175 [42], Slo1/BK channels [24,26] and TWIK2 [43]. While efflux of monovalent cations can support the acidification of lysosomes [39], the presence of TMEM175 is important for pH stability under starving conductions in RAW 264.7 macrophages [42]. Slo1/BK channels were proposed to provide charge compensation for the uptake and release of lysosomal Ca^2+^, respectively [24,26].

Chloride (Cl^−^) is the most abundant anion in lysosomes with a luminal concentration of up to 120 mM [44,45]. While the function of Cl^−^ in providing the electrical shunt in endosomal acidification is well accepted, its role as a counterion for the acidification of lysosomes is still a matter of debate [19,46]. The anion transport protein ClC-7 provides the main lysosomal Cl^−^ conductance [47,48,49]. Like the other vesicular CLCs [50,51,52], ClC-7 functions as a voltage-dependent, outwardly rectifying Cl^−^/H^+^-exchanger coupling the counter-transport of one proton to two chloride ions per transport cycle [49,53,54]. Loss of ClC-7 or its obligate β-subunit Ostm1 [54,55] impairs lysosomal protein degradation [56] and leads to a neurodegenerative lysosomal storage disease and osteopetrosis, likely resulting from lysosomal dysfunction, in mice and humans [47,48,55,57,58]. A mouse model with a ClC-7 mutation that uncouples Cl^−^ transport from H^+^ counter-transport displays the same lysosomal pathology like ClC-7-deficient mice and defective bone resorption [53]. The lysosomal Cl^−^ concentration is reduced due to loss of pH gradient-driven Cl^−^ accumulation in cells from mouse models lacking ClC-7 or expressing the uncoupling mutant, while lysosomes are normally acidified [48,53]. This correlation of reduced lysosomal Cl^−^ concentration with lysosomal dysfunction independent of normal acidification [45] hints towards the role of luminal Cl^−^ in lysosomal function, either directly or via its effect on lysosomal ion homeostasis in general [46]. Surprisingly, not only loss-of-function mutations but also mutations that accelerate the normally slow voltage-dependent activation of ClC-7 were found to underlie osteopetrosis [54,59,60]. These dysfunctions were speculated to be linked to voltage jumps during lysosomal Ca^2+^ release [54], but the exact mechanisms by which altered kinetics impinge on lysosomal function are still unknown.

Despite increasing knowledge about lysosomal ion transporters, relatively little is known about their combined effect on organellar ion homeostasis as a whole. So far, mathematical models of organellar pH regulation allowed simulating ion homeostasis in endosomes and lysosomes [53,61,62,63,64]. However, even though one of the previously published models incorporates a semi-calibrated description of the ClC-7 antiporter [62], it lacks essential components of lysosomal physiology such as the slow voltage-gated activation of ClC-7 and the uptake and release of Ca^2+^. A mathematical model of resorption lacuna acidification refers to the exocytosis of lysosomes as a Ca^2+^-mediated process, yet it does not include Ca^2+^, nor does it explicitly consider the lysosomal compartment [65]. As the triggered release of Ca^2+^ from lysosomes may lead to voltage jumps, we hypothesize that the lysosomal Ca^2+^ dynamics may be altered by pathogenic ClC-7 mutations that enhance the voltage-dependent activation kinetics of the chloride/proton exchanger. To explore the impact of ClC-7 on lysosomal Ca^2+^ dynamics we developed a mathematical model that offers a mechanistic description for the role of ClC-7 on lysosomal Ca^2+^ uptake and release. We considered four different ClC-7 scenarios (wild-type, fast, uncoupled, and knock-out) to additionally investigate different levels of chloride transport disruption. Our findings show subtle differences between the different simulated ClC-7 activation kinetics and further suggest a previously neglected important role for ClC-7 in lysosomal Ca^2+^ dynamics.

## 2. Materials and Methods

### 2.1. Model Design

To investigate the putative differential effect of chloride transport on lysosomal ion homeostasis, we generated an ODE mathematical model for this system. Our mathematical model builds upon a previously published model for lysosomal homeostasis [61,62], and further includes the (de)activation kinetics of the ClC-7 antiporter and Ca^2+^ uptake/release mechanisms. Our new model tracks the total number of ions within the lysosomal lumen over time. It considers different types of lysosomal ion channels and exchangers and two possible lysosomal Ca^2+^ transporters. The variation in the total number of each ion within the lysosome is described by an ordinary differential equation (ODE), and the rate of change is determined by the flux of the corresponding ion across the lysosomal membrane. The model contains 36 variables and 33 parameters, as listed in Tables S1 and S2. The parameters were mainly taken from published experimental data. This information is specified in Table S1, which contains all parameters and the corresponding references. The only parameter that was adjusted in each simulation was the Ca^2+^ permeability P_Ca_^2+^.

The model considers different elements affecting lysosomal ion homeostasis, among which are: (i) the V-ATPase pump, (ii) a proton leak, (iii) the luminal proton buffering capacity, (iv) ClC-7 chloride/proton exchanger, (v) Ca^2+^/proton exchanger (CAX), (vi) passive channels for K^+^, Na^+^, and Ca^2+^, and (vii) Donnan particles, which are negatively charged particles or molecules trapped in the lysosomal lumen. It allows for the simulation of different scenarios mimicking the differential transport of chloride, its impact on Ca^2+^ uptake and release and ultimately on lysosomal homeostasis. To investigate the possible differential impact of chloride dynamics on lysosomal acidification, we simulated four different ClC-7 scenarios:

1. ClC-7^WT^, which mimics a slowly voltage-gated antiporter [54] with delayed—not instantaneous—(de)activation kinetics.
2. ClC-7^fast^, which mimics a ClC-7 antiporter with instantaneous (de)activation. This is an extreme scenario of the experimental observations, in which mutations accelerating the (de)activation kinetics also led to osteopetrosis [54].
3. ClC-7^unc^, in which the chloride transport is mimicked by a passive chloride flux through a channel-like ClC-7 antiporter.
4. ClC-7^ko^, which represents the absence of the antiporter.

**Figure 1:**
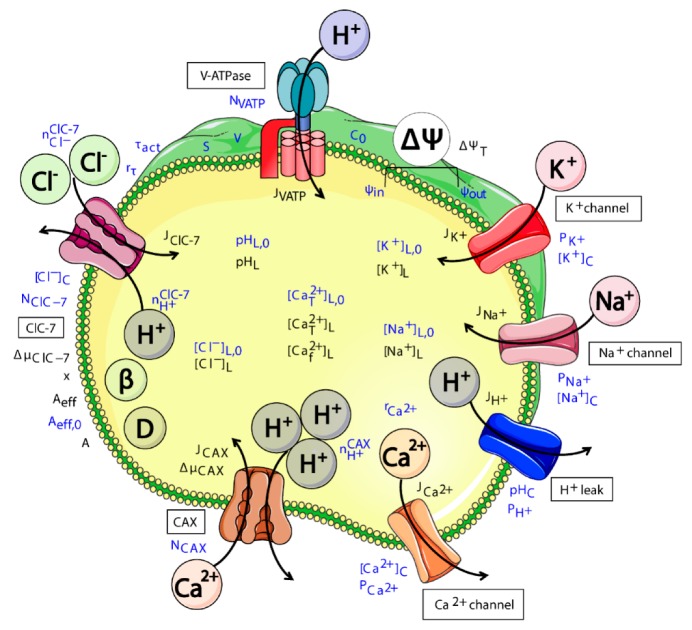
Schematic representation of all components included in the model of ion homeostasis. The V-ATPase pumps protons (H^+^) into the lysosomal lumen for acidification. ClC-7 antiporter transports chloride ions (Cl^−^) in exchange for protons. CAX transports calcium ions (Ca^2+^) in exchange for protons. Proton (H^+^), potassium (K^+^), sodium (Na^+^) and Ca^2+^ channels allow the passive movement of these ions. The Donnan particles (D) affect lysosomal acidification through the membrane potential, and buffering capacity (β) of the lumen affects the rate of pH changes. The names of the channels and transporters are indicated with labels. Variables and parameters are written in blue and black, respectively. The flux across the membrane irrespective of direction is represented by black arrows. The cartoon was created using Servier Medical Art templates (https://smart.servier.com), which are licensed under a Creative Commons License (https://creativecommons.org/licenses/by/3.0/).

The model was implemented in Matlab^TM^ 2016b with the solver ODE15s (with minimum time-step size 10^−13^ s, same units as for the parameters in the corresponding simulation, and maximum step size (10% of the total time span) as default). The absolute and relative tolerances were set to 10^−6^. The complete mathematical description of the model is provided in Supplementary Materials, and a schematic representation of the model is provided in Figure 1. The model is available at BioModels (http://www.ebu.ac.uk/biomodels).

### 2.2. Sensitivity Analysis

We investigated the sensitivity of our model by varying every single input parameter (Table S1) by ±10%. For each simulation, we analysed disturbances on the steady-state output values of luminal pH, luminal concentrations of protons, potassium, sodium, chloride, free Ca^2+^, total Ca^2+^, and membrane potential. For this, we calculated the relative difference between the output value obtained from the test simulation (with variation) and from the reference simulation (without variation, Supplementary Materials). The luminal pH presented relative differences lower than 2% for every test, meaning that this variable was robust against changes in all input parameters (Figure S9). The largest disturbances where found for luminal concentrations of potassium, sodium chloride, and calcium ions when the initial value of cytosolic pH was varied, and for luminal proton concentration when the initial value of luminal pH was varied.

## 3. Results

### 3.1. In Silico Simulations Recapitulate Differential Voltage-Dependent Clc-7 Activation Kinetics

In our model, we aimed at providing a mathematical description that accurately represents the differential voltage-dependent ClC-7 activation kinetics. Electrophysiological measurements of ClC-7, targeted to the plasma membrane by disruption of lysosomal sorting motifs [66], revealed that this transporter mediates outwardly rectifying (i.e., preferential transport of chloride into the cytosol) Cl^−^/H^+^ exchange, which is slowly gated by voltage changes [54]. While a previously published model of lysosomal ion homeostasis considered the outward rectification of the ClC-7 antiporter, it did not take into account the time-dependence of its voltage gated activation [62]. Instead, the mathematical description for the ClC-7 turnover rate is time-independent and represents an instantaneous (de)activation kinetics [62]. Therefore, we used this formulation to mimic the turnover rate of an extremely fast ClC-7 (ClC-7^fast^), and introduced a slight modification in the equation in order to have an explicit term for the activity, which was then used to model the non-instantaneous (de)activation kinetics of strong outwardly rectifying currents for the wild-type ClC-7. Thus, we describe the ClC-7^fast^ turnover rate (J_ClC-7_^fast^) as

(1) $$J_{\mathrm{ClC}\text{-}7^{\mathrm{fast}}}=N_{\mathrm{ClC}\text{-}7}\cdot A\cdot \Delta \mu_{\mathrm{ClC}\text{-}7}$$

N_ClC-7_ is the number of ClC-7 antiporters, Δ$\mu_{\mathrm{ClC}\text{-}7}$ is the driving force for the turnover (Equation (S30) in Supplementary Materials), and A is the activity of the ClC-7 antiporter, which includes the rectification:

(2) $$A=0.3\cdot x+1.5\cdot 10^{-5}\cdot \left(1-x\right)\cdot \Delta \mu_{\mathrm{ClC}\text{-}7}^{2}$$

The switching function x varies from zero at negative membrane potentials (Δ*ψ*) to 1 at positive Δ*ψ* (Equation (S32) in Supplementary Materials). We considered the voltage at the cytosol to be zero for all simulations. Thus, the activity (A) is proportional to the square of the ClC-7 driving force (Δ$\mu_{\mathrm{ClC}\text{-}7}$) at negative Δψ and reaches a maximum value of 0.3 at positive Δψ.

To mimic the (de)activation kinetics of the wild-type ClC-7 (ClC-7^WT^) we described the time-dependent ClC-7^WT^ turnover rate (J_ClC-7_^WT^) in terms of the effective activity (A_eff_) that the antiporter is able to achieve at a specific time:

(3) $$J_{\mathrm{ClC}\text{-}7^{\mathrm{WT}}}={\;N}_{\mathrm{ClC}\text{-}7}\cdot A_{\mathrm{eff}}\cdot \Delta \mu_{\mathrm{ClC}\text{-}7}$$

A_eff_ varies in time according to:

(4) $$\frac{{\mathrm{dA}}_{\mathrm{eff}}}{\mathrm{dt}}=\frac{1}{\tau }\left(A-A_{\mathrm{eff}}\right)$$

Hence, if A is higher (lower) than A_eff_, then A_eff_ increases (decreases) according to the activation (deactivation) time τ = τ_act_ (τ = τ_deact_) until it reaches the value of A (for a detailed explanation, see also Equation (S34) in the Supplementary Materials). For simplicity, and in agreement with experimental data on various ClC-7 mutants with altered (de)activation kinetics [60], we considered the deactivation time τ_deact_ to be proportional to the activation time τ_act_

(5) $$\tau_{\mathrm{deact}}=\tau_{\mathrm{act}}\cdot r_{\tau }$$

where r_τ_ is the deactivation-to-activation ratio set to 0.25, as the deactivation time was found to be around one quarter of the activation time [67].

For a fixed value of membrane potential, the chloride current through ClC-7^WT^ is equal to the chloride current through ClC-7^fast^ after a certain time (depending on the experimental conditions). Hence, the turnover rate of ClC-7^fast^ antiporter is—from time zero—equal to the steady-state value (reached after a certain time) of the turnover rate of the ClC-7^WT^. Thus, ClC-7^fast^ corresponds to a “steady-state antiporter”. Since we aimed at simulating the difference between the slowly voltage-gated ClC-7 (ClC-7^WT^) and a fast mutant, we chose the “steady-state antiporter” as an extreme example of the fast mutant (ClC-7^fast^).

**Figure 2:**
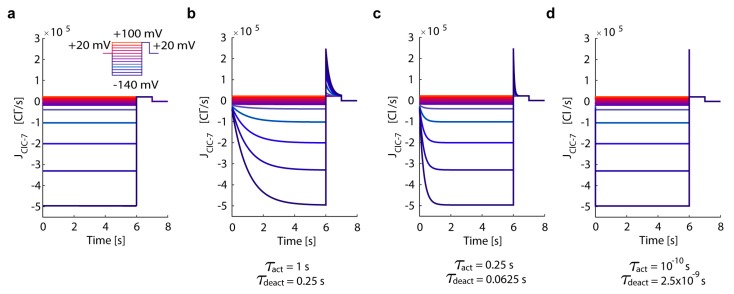
In silico simulation of voltage-clamp traces of ClC-7. Starting from a resting potential of +20 mV, we simulated a pulse protocol from −140 mV to +100 mV in 20-mV steps for 6 s, followed by +100 mV for 1 s, after returning to the resting potential. Depicted are the turnover rates for (**a**) ClC-7^fast^ antiporter, and (**b**) ClC-7^WT^ antiporter with relatively slow (de) activation kinetics (τ_act_ = 1 s), (**c**) a ClC-7 antiporter with moderately accelerated (de) activation kinetics (τ_act_ = 0.25 s), and (**d**) a ClC-7 antiporter with an extremely accelerated (de) activation kinetics (τ_act_ = 10^−10^ s). The colour gradient varies from dark blue for negative voltages to red for positive voltages (cytosolic potential defined as zero) as indicated in the pulse protocol (inset in (**a**)).

To illustrate the differences between the fast and wild-type ClC-7 and investigate the impact of (de)activation times, we simulated a voltage-clamp experiment, considering a ClC-7^fast^, ClC-7^WT^, and a ClC-7 with different (de)activation times. To recapitulate previously published experimental observations of ClC-7-mediated currents [54,62], we simulated the proportional underlying turnover rates of ClC-7. We took voltage pulses starting from an extra-cytosolic resting potential of +20 mV and ranging from −140 mV to +100 mV in 20-mV steps for 6 s, followed by +100 mV for 1 s, before returning to the resting potential (Figure 2).

Our mathematical description of the ClC-7 activation kinetics (activation time τ_act_ = 1 s retrieved from Leisle et al. [54], deactivation time τ_deact_ = 0.25 s of one quarter of the activation time as reported by Ludwig et al. [67], Table S1) allows for the simulation of wild-type-like currents traces in agreement with experimental observations [54,60,62]. Importantly, by decreasing τ_act_ (and therefore also τ_deact_) we were able to mimic current traces of a ClC-7 with accelerated kinetics. In particular, by setting a very short (de)activation time for ClC-7 (τ_act_ = 10^−10^ s, τ_deact_ = 2.5 × 10^−9^ s) we recapitulated the behaviour of ClC-7^fast^. Thus, our mathematical description of the ClC-7 activation kinetics allows for the simulation of wild-type-like currents traces in agreement with experimental observations.

### 3.2. ClC-7 Activation Kinetics Do Not Affect Lysosomal Acidification

To investigate the impact of altered chloride transport on lysosomal acidification, we simulated four different ClC-7 scenarios: (i) ClC-7^WT^, a wild-type ClC-7 representing the slowly-voltage gated antiporter as experimentally described [54]; (ii) ClC-7^fast^, a fast ClC-7 which mimics a ClC-7 antiporter with instantaneous (de)activation kinetics. This is an extreme case of the acceleration experimentally observed for some osteopetrosis-causing mutations [54,59,60]; (iii) ClC-7^unc^, in which chloride transport is uncoupled from proton counter-transport, rendering ClC-7 a pure chloride conductance with linear voltage-dependence and instantaneous (de)activation [53,54]; (iv) ClC-7^ko^, the knockout of ClC-7, which represents the complete absence of the antiporter.

The time-dependent variation in the number of luminal chloride ions varies with the above-described scenarios as follows:

(6) $$\frac{{\mathrm{dNCl}}^{-}}{\mathrm{dt}}=\{\begin{array}{l} n_{{\mathrm{Cl}}^{-}}^{\mathrm{ClC}\text{-}7}\cdot J_{\mathrm{ClC}\text{-}7^{\mathrm{WT}}}\\ n_{{\mathrm{Cl}}^{-}}^{\mathrm{ClC}\text{-}7}\cdot J_{\mathrm{ClC}\text{-}7^{\mathrm{fast}}}\\ J_{\mathrm{ClC}\text{-}7^{\mathrm{unc}}}\\ J_{\mathrm{ClC}\text{-}7^{\mathrm{ko}}} \end{array}\;\begin{array}{c} {\;,\;\mathrm{for}\;\mathrm{ClC}\text{-}7}^{\mathrm{WT}}\\ {\;,\;\mathrm{for}\;\mathrm{ClC}\text{-}7}^{\mathrm{fast}}\\ {\;,\;\mathrm{for}\;\mathrm{ClC}\text{-}7}^{\mathrm{unc}}\\ {\;,\;\mathrm{for}\;\mathrm{ClC}\text{-}7}^{\mathrm{ko}} \end{array}$$

where $n_{{\mathrm{Cl}}^{-}}^{\mathrm{ClC}\text{-}7}$ is the Cl^−^/H^+^ stoichiometry of ClC-7; and J_ClC-7_^WT^, J_ClC-7_^fast^, J_ClC-7_^unc^, and J_ClC-7_^ko^ are the respective ClC-7 turnover rates (positive for chloride influx).

**Figure 3:**
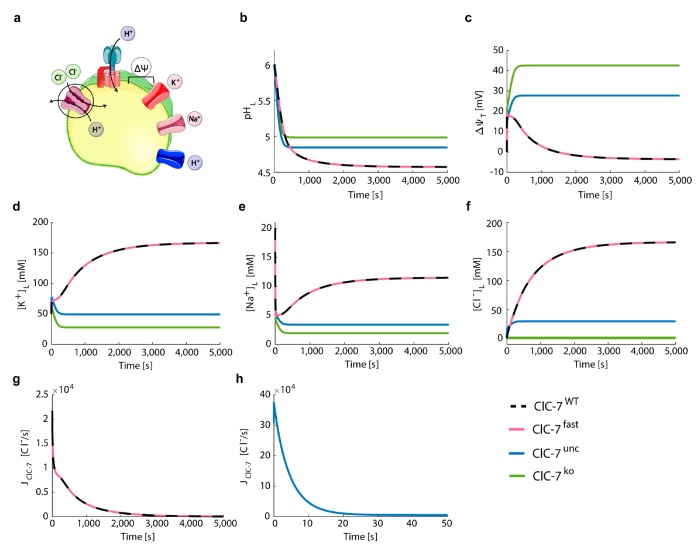
Differences in ClC-7 kinetics do not affect lysosomal acidification. (**a**) Schematic representation of the model with ClC-7 antiporters, V-ATPases, potassium and sodium channels, and proton leak. The cartoon was created using Servier Medical Art templates (https://smart.servier.com), licensed under a Creative Commons License (https://creativecommons.org/licenses/by/3.0/). (**b**–**h**) Depicted for the different ClC-7 scenarios during lysosomal acidification (ClC-7^WT^, dashed black line; ClC-7^fast^, red; ClC-7^unc^, blue; ClC-7^ko^, green) are (**b**) luminal pH, (**c**) total membrane potential, (**d**) luminal concentrations of potassium, (**e**) sodium, and (**f**) chloride ions, as well as the turnover rates of (**g**) ClC-7^WT^ and ClC-7^fast^, and (**h**) ClC-7^unc^. Initial conditions provided in Table S1.

We simulated the uncoupled transport of chloride and protons as a passive chloride flux through a “channel-like” ClC-7 as previously defined by Ishida et al. [62]. Therefore, we describe the ClC-7^unc^ turnover rate using the equation from Ishida et al. [62]

(7) $$J_{\mathrm{ClC}\text{-}7^{\mathrm{unc}}}=P_{{\mathrm{Cl}}^{-}}\cdot S\cdot \frac{U}{1-e^{-U}}\cdot \left({\left[{\mathrm{Cl}}^{-}\right]}_{e}-{\left[{\mathrm{Cl}}^{-}\right]}_{i}\cdot e^{-U}\right)\cdot \frac{N_{A}}{10^{3}}$$

where $P_{{\mathrm{Cl}}^{-}}$ is the permeability per unit area for chloride ions, S is the lysosome surface area, N_A_ is the Avogadro’s number, U = (Δ*ψ*·F)/(R·T) is the reduced membrane potential as previously formulated [62], [Cl^−^]_e_ and [Cl^−^]_i_ are the cytosolic and luminal chloride concentration modified by a Boltzmann factor, respectively (Equations (S5) and (S6) in Supplementary Materials). We determined the turnover rate for ClC-7^ko^ (J_ClC-7_^ko^) with Equation (3) by setting N_ClC-7_ = 0. The initial luminal concentrations of K^+^, Na^+^, and Cl^−^ concentrations were set to 50 mM, 20 mM, and 1 mM, respectively, as reported by Steinberg et al. [39] corresponding to a non-acidic lysosome. We simulated the acidification of a lysosome containing V-ATPase pumps, channels for potassium and sodium, a proton leak (passive flow of H^+^) and either of the different types of ClC-7 antiporters (Figure 3).

The simulation of the wild-type and fast ClC-7 scenarios yielded the most acidic luminal pH (pH_L_ = 4.6, Figure 3b), a slightly luminal-negative total membrane potential (Δ*ψ*_T_ = −3.4 mV, Figure 3c), and the highest luminal potassium, sodium, and chloride concentrations ([K^+^]_L_ = 167 mM, [Na^+^]_L_ = 12 mM, and [Cl^−^]_L_ = 166 mM, Figure 3d–f). As previously shown experimentally [53,62], the absence of a Cl^−^/H^+^ exchanger (ClC-7^unc^ and ClC-7^ko^) leads to a less acidic pH. In the ClC-7^ko^ scenario, which mimics ClC-7 deficiency, we obtained the least acidification (pH_L_ = 5) and the highest total membrane potential (Δ*ψ*_T_ = 42.6 mV). The ClC-7^unc^ led to a steady-state pH_L_ of 4.9 and a positive total membrane potential (Δ*ψ*_T_) of 27.8 mV (Figure 3b,c). For both ClC-7^unc^ and ClC-7^ko^ we observed a reduction of luminal potassium ([K^+^]_L_)and sodium ([Na^+^]_L_), since they served as counter ions supporting acidification (Figure 3d,e). While the luminal concentration of chloride was constant for the ClC-7^ko^ scenario ([Cl^−^]_L_ = [Cl^−^]_L,0_ = 1 mM) because the only possible chloride transport pathway (ClC-7) was absent and remained low in our simulations of ClC-7^unc^ ([Cl^−^]_L_ = 29.6 mM), [Cl^−^]_L_ increased to 166 mM for the ClC-7^WT^ and ClC-7^fast^ (Figure 3f). Chloride transport through ClC-7^WT^ (and ClC-7^fast^) lasted for 5000 s (Figure 3g), whereas chloride influx through the antiporter occurred during the first 40 s of the acidification process (Figure 3h).

Our data corroborate the notion that perturbations in chloride transport across the lysosomal membrane lead to differences in acidification and in the steady-state luminal ion concentrations. In addition, we observed no differences in lysosomal acidification between ClC-7^fast^ and ClC-7^WT^, since the values for the ClC-7 driving force reached during acidification did not induce changes in the activity of the ClC-7 antiporter (Supplementary Materials).

### 3.3. Perturbations on ClC-7 Differentially Affect Ca^2+^ Release

Differences in voltage dependent ClC-7 kinetics were hypothesized to be relevant during voltage jumps associated with lysosomal Ca^2+^ release [54]. To investigate whether differential chloride transport within the different ClC-7 scenarios impacts on Ca^2+^ release, we simulated the opening of Ca^2+^ channels from the steady-state conditions obtained in Figure 3 (Table S3). As several Ca^2+^ channels with diverse biophysical properties and different voltage- and pH-dependencies have been reported (e.g., TRPML1, TPC2, P2X4, VGCC). Implementing all these possibilities would unavoidably increase the complexity of the model. This would restrict the detailed analysis of compensation mechanisms in perturbed scenarios. For simplicity, we opted to simulate the release channels solely via Ca^2+^ permeability.

The change in total Ca^2+^ is described as

(8) $$\frac{{\mathrm{dNCa}}_{T}^{2+}}{\mathrm{dt}}=J_{{\mathrm{Ca}}^{2+}}$$

where J_Ca_^2+^ is the passive flow through Ca^2+^ channel (positive for Ca^2+^ influx)

(9) $$J_{{\mathrm{Ca}}^{2+}}=P_{{\mathrm{Ca}}^{2+}}\cdot S\cdot \frac{2U}{1-e^{-2U}}\cdot \left({\left[{\mathrm{Ca}}_{f}^{2+}\right]}_{e}\cdot e^{-2U}-{\left[{\mathrm{Ca}}_{f}^{2+}\right]}_{i}\right)\cdot \frac{N_{A}}{10^{3}}$$

P_Ca_^2+^ is the permeability per unit area for calcium ions, ([Ca^2+^_f_]_i_ and [Ca^2+^_f_]_e_) are the modified luminal and cytosolic free Ca^2+^ concentration, respectively (Equations (S11) and (S12) in Supplementary Materials).

**Figure 4:**
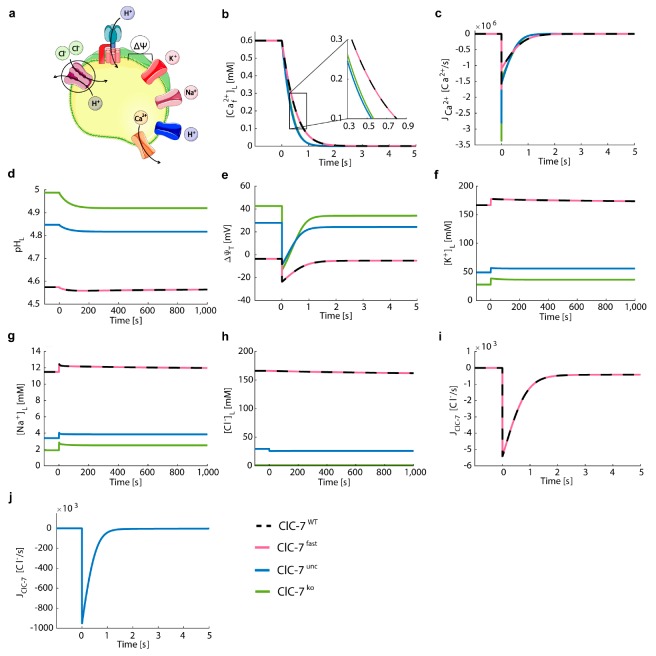
The cation channels Na^+^ and K^+^ neutralize the influence of ClC-7 on Ca^2+^ release. (**a**) Schematic representation of the model with ClC-7 antiporters, V-ATPases, potassium and sodium channels, proton leak, and Ca^2+^ release channel. The cartoon was created using Servier Medical Art templates (https://smart.servier.com), licensed under a Creative Commons License (https://creativecommons.org/licenses/by/3.0/). (**b**–**j**) Depicted for the different ClC-7 scenarios during triggered Ca^2+^ release (ClC-7^WT^, dashed black line; ClC-7^fast^, red; ClC-7^unc^, blue; ClC-7^ko^, green) are (**b**) luminal free Ca^2+^ concentration, (**c**) Ca^2+^ flux, (**d**) luminal pH, (**e**) total membrane potential, (**f**) luminal concentrations of potassium, (**g**) sodium and (**h**) chloride ions, as well as the turnover rates of (**i**) ClC-7^WT^ and ClC-7^fast^, and (**j**) ClC-7^unc^. The initial conditions were set to the steady-state values of Figure 3 (Supplementary Table S3). From t = 0 s, the lysosomal membrane was permeable to calcium ions (*P_Ca_*^2+^ = 8.9 × 10^−5^ cm/s).

We adjusted the Ca^2+^ permeability in order to achieve an arbitrary 10-fold decrease of luminal free Ca^2+^ concentration within 1 s for ClC-7^WT^, and we used the same value of Ca^2+^ permeability (P_Ca_^2+^ = 8.9 × 10^−5^ cm/s) to simulate the other ClC-7 scenarios (Figure 4). The value given to the permeability does not have an impact on the relative differences between the ClC-7 scenarios, as the steady-state values of luminal pH, ionic concentrations and membrane potentials remain unchanged.

We observed an efficient Ca^2+^ release in all scenarios (Figure 4b), accompanied by small acidification (Figure 4d). Emptying of the lysosome was slightly faster for ClC-7^ko^ and ClC-7^unc^. In these scenarios, the maximum Ca^2+^ efflux was stronger (J_Ca_^2+^ = −3.4 × 10^−6^ and −2.8 × 10^−6^ Ca^2+^/s, respectively) than the efflux observed for ClC-7^WT^ and ClC-7^fast^ (J_Ca_^2+^ = −1.7 × 10^−6^ Figure 4c). No differences were observed between ClC-7^fast^ and ClC-7^WT^ (Supplementary Materials). The maximum chloride turnover rate for the ClC-7^WT^ and ClC-7^fast^ were about three orders of magnitude lower compared to ClC-7^unc^ (J_ClC-7_^WT^ = J_ClC-7_^fast^ = −5.4 × 10^3^ Cl^−^/s versus J_ClC-7_^unc^ = −950 × 10^3^ Cl^−^/s) (Figure 4i,j), causing the delayed Ca^2+^ release in the ClC-7^WT^ and ClC-7^fast^ scenarios. This led to a decrement in chloride concentration of 2.4% and 12% for ClC-7^WT^ and ClC-7^unc^, respectively. While in the ClC-7^WT^, ClC-7^fast^ and ClC-7^unc^ scenarios chloride contributed, together with the other ions, to compensate for the Ca^2+^ release, in the ClC-7^ko^ scenario the Ca^2+^ release was compensated only by the influx of proton, potassium, and sodium (Figure 4d,f–h, respectively). For the ClC-7^ko^, the luminal concentrations of potassium (${\left[K^{+}\right]}_{L}$) and sodium $({\left[{\mathrm{Na}}^{+}\right]}_{L}$) ions increased circa 30%, during Ca^2+^ release. For ClC-7^unc^, the increase in luminal potassium and sodium concentrations was about 14% and 15%, respectively, and for the wild-type and fast scenarios only 4% (Figure 4f,g). We observed the same temporal variation in the values of luminal free Ca^2+^ concentration ([Ca^2+^_f_]_L_) for ClC-7^WT^ and ClC-7^fast^ scenarios. The opening of the Ca^2+^ channel did not result in changes of the ClC-7 driving force (dependent on voltage, pH and Cl^−^ gradient, see Figure S1) large enough to alter the activity of the ClC-7 antiporter. Hence, during Ca^2+^ release, the kinetics have no impact on their (ClC-7^fast^ vs. ClC7^WT^) activities.

As an exemplary alternative Ca^2+^ release pathway, we simulated the Ca^2+^ permeability with voltage- and pH-dependence as previously described for the prominent lysosomal Ca^2+^ release channel TRPML1 [68,69] (Figure S2). We repeated the simulation of Figure 4, but considering a channel similar to TRPML1 as the only Ca^2+^ pathway and therefore the change in total calcium ions is described only by the flux through the voltage- and pH-dependent channel (${\mathrm{dNCa}}_{T}^{2+}/\mathrm{dt}=J_{\mathrm{TRPML}1}$). The mathematical description of this flux, $J_{\mathrm{TRPML}1}$, is provided in the Supplementary Materials (Equations (S43)–(S45)). While we observed some quantitative differences between the TRPML1-like permeability (Figure S2) and the voltage- and pH-independent Ca^2+^ channel (Figure 4), the relative differences between the ClC-7 scenarios remained unaltered. The steady-state values of luminal pH, ionic concentrations, and membrane potential were the same for both Ca^2+^ permeabilities.

Next, we evaluated the effect of ClC-7 in Ca^2+^ efflux in the absence of potassium and sodium ions (P_K_^+^ = P_Na_^+^ = 0). In this case, only proton influx and chloride efflux can provide the required counter-ion transport, resulting in a slower Ca^2+^ release (Figure S3). The Ca^2+^ efflux during the first second of the simulation was higher for the uncoupled than for the other scenarios, leading to a faster Ca^2+^ release. Similarly, we observed slightly increased values of Ca^2+^ release for the fast compared to the wild-type scenario. These small differences were due to the strongly negative ClC-7 driving forces induced during Ca^2+^ efflux, which led to a change in the ClC-7 activity from A = 0.3 to A = 0.57 (Supplementary Materials), and therefore to an instantaneous versus slow activation of the fast and WT scenarios, respectively.

### 3.4. Chloride/Proton Exchanger Supports Lysosomal Ca^2+^ Uptake

The mechanisms of lysosomal Ca^2+^ uptake are enigmatic [34]. We tested two possibilities: (i) refilling from the cytosol via Ca^2+^/H^+^ exchange (which we refer to as CAX, although this may be mediated by any protein complex, not necessarily belonging to the CAX protein family) [28,30,31] with variable stoichiometries, or (ii) refilling from the Ca^2+^-rich endoplasmic reticulum (ER) via Ca^2+^ channels [32,33,34].

To describe the change in total calcium ions by the combination of CAX and Ca^2+^ channels (or Ca^2+^ leak) we reformulated Equation (8), which described solely the Ca^2+^ leak type of pathway, as:

(10) $$\frac{{\mathrm{dNCa}}_{T}^{2+}}{\mathrm{dt}}=n_{{\mathrm{Ca}}^{2+}}^{\mathrm{CAX}}\cdot J_{\mathrm{CAX}}+J_{{\mathrm{Ca}}^{2+}}$$

The turnover rate of CAX (positive for Ca^2+^ influx) is described as:

(11) $$J_{\mathrm{CAX}}=N_{\mathrm{CAX}}\cdot \Delta \mu_{\mathrm{CAX}}$$

where $N_{\mathrm{CAX}}$ is the number of CAX and $\Delta \mu_{\mathrm{CAX}}$ is the driving force for the CAX antiporter:

(12) $$\Delta \mu_{\mathrm{CAX}}=(n_{H^{+}}^{\mathrm{CAX}}-2\cdot n_{{\mathrm{Ca}}^{2+}}^{\mathrm{CAX}})\cdot \Delta \psi +\frac{\mathrm{RT}}{F}\left(2.3\cdot n_{H^{+}}^{\mathrm{CAX}}\cdot ({\mathrm{pH}}_{e}-{\mathrm{pH}}_{i})+\frac{n_{{\mathrm{Ca}}^{2+}}^{\mathrm{CAX}}}{2}\cdot \ln\frac{{\left[{\mathrm{Ca}}_{f}^{2+}\right]}_{i}}{{\left[{\mathrm{Ca}}_{f}^{2+}\right]}_{e}}\right)$$

$n_{H^{+}}^{\mathrm{CAX}}$ and $n_{{\mathrm{Ca}}^{2+}}^{\mathrm{CAX}}$ are the CAX stoichiometries for protons and Ca^2+^, respectively, and ${\mathrm{pH}}_{e}$ and ${\mathrm{pH}}_{i}$ are the Boltzmann-modified cytosolic and luminal pH, respectively (Equations (S3) and (S4) in Supplementary Materials).

We first performed test simulations for the wild-type ClC-7 scenario in order to calibrate the number and stoichiometry of CAXs. We simulated the Ca^2+^ uptake via CAX from the steady-state conditions of Figure 3 (i.e., after Ca^2+^ release, Supplementary Table S3), with a cytosolic Ca^2+^ concentration of 100 nM, and zero Ca^2+^ permeability ($P_{{\mathrm{Ca}}^{2+}}=0$). The calibrations were performed for 1, 10, 20 and 30 CAX with exchange stoichiometries of 1H^+^:1Ca^2+^, 2H^+^:1Ca^2+^, and 3H^+^:1Ca^2+^. While the 2H^+^:1Ca^2+^ stoichiometry does not lead to a net charge transfer, the 1H^+^:1Ca^2+^ and 3H^+^:1Ca^2+^ are electrogenic with opposing current directions.

**Figure 5:**
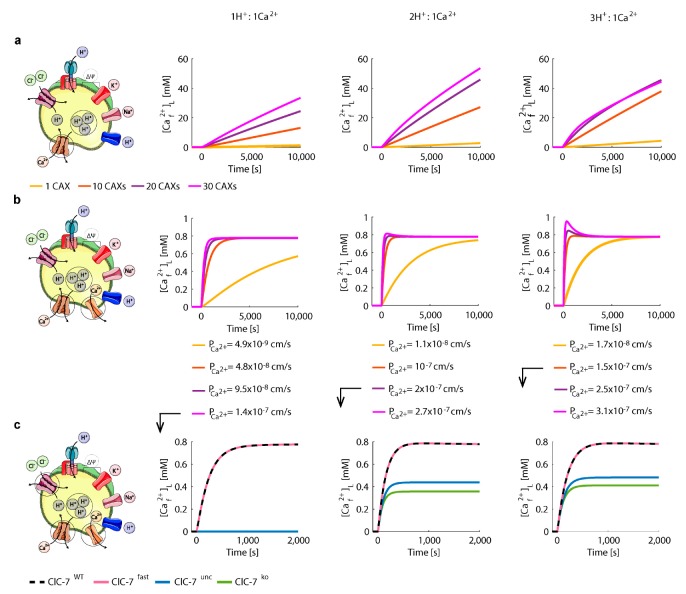
Ca^2+^/H^+^ exchange mediates effective Ca^2+^ uptake in the presence of a Ca^2+^ leak. (**a**,**b**) Simulations of Ca^2+^ uptake via CAX with three different stoichiometries as depicted (1:1, 2:1, 3:1) in (**a**) absence and (**b**) presence of Ca^2+^ leak for wild-type ClC-7. The luminal free Ca^2+^ concentrations are shown for 1, 10, 20 and 30 CAXs. (**b**) For each case, the Ca^2+^ permeability was set to enable a steady-state luminal free Ca^2+^ concentration. (**c**) Simulations for the four ClC-7 scenarios considering different CAX configurations: 30 CAX, 1H^+^:1Ca^2+^; 20 CAX, 2H^+^:1Ca^2+^, and 10 CAX, 3H^+^:1Ca^2+^, using the same Ca^2+^ permeability as in (**b**) for all ClC-7 scenarios. The initial conditions were set to the steady-state values of Figure 4 (i.e., after Ca^2+^ release, Supplementary Table S3). The cartoons were created using Servier Medical Art templates (https://smart.servier.com), licensed under a Creative Commons License (https://creativecommons.org/licenses/by/3.0/).

Ca^2+^ refilling via CAX led to a continuous uptake, without reaching a steady state during the simulation time (Figure 5a). Remarkably, when adding a passive Ca^2+^ leak, which may be mediated by Ca^2+^ channels, and adjusting it for each CAX condition, we were able to reach a physiological steady-state for luminal free Ca^2+^ (Figure 5b).

As expected, for increasing numbers of CAX (i.e., increasing Ca^2+^ uptake), we required higher values of Ca^2+^ permeability ($P_{{\mathrm{Ca}}^{2+}}$). For 1 CAX the ion homeostasis was reached only after 2 h, and therefore it was excluded. We observed that the impact of the number of CAX on luminal pH (${\mathrm{pH}}_{L}$), total membrane potential ($\Delta \psi_{T}$), luminal ion concentrations (${\left[{\mathrm{Na}}^{+}\right]}_{L}$, ${\left[K^{+}\right]}_{L},\;{\left[{\mathrm{Cl}}^{-}\right]}_{L}$) and ClC-7 turnover rate ($J_{\mathrm{ClC}\text{-}7}$) was higher for larger H^+^/Ca^2+^ ratios (Figure S4). As lysosomes have an acidic luminal pH (${\mathrm{pH}}_{L}$) of 4.5—5 [19], the configurations of 20 and 30 CAX with 3:1 stoichiometry were excluded as they lead to luminal pH outside this range. Hence, the configurations of 10 CAX with 3:1, 20 CAX with 2:1, and 30 CAXs with 1:1 stoichiometry were included for further analysis.

The Ca^2+^ uptake for the four ClC-7 scenarios for the selected CAX configurations is depicted in Figure 5c and variations in other lysosomal elements (pH, membrane potential, concentrations of cation and chloride) are depicted in Figures S5–S7. No differences were observed between the wild-type and fast scenarios, which reached the highest steady-state value of luminal free Ca^2+^ concentration (${\left[{\mathrm{Ca}}_{f}^{2+}\right]}_{L}$ = 0.78 mM). In contrast, the uncoupled and the knockout scenarios reached lower steady-state Ca^2+^ concentrations. With CAXs of 3:1 and 2:1 H^+^/Ca^2+^ stoichiometry, these lysosomes accumulated about half the Ca^2+^ concentration as compared to wild-type ClC-7, whereas with the CAX of 1:1 stoichiometry lysosomes did not accumulate Ca^2+^ at all (Figure 5c). With this 1:1 stoichiometry, the contribution of the luminal-positive membrane potential in the uncoupled and knockout scenarios prevented Ca^2+^ uptake, and hence to no change in the other lysosomal elements (Figure S7).

Interestingly, with 2:1 stoichiometry we did not observe the initial chloride efflux through the uncoupled ClC-7 (Figure S6) presented in the 3:1 stoichiometry scenario (Figure S5). This could be because for the 3:1 stoichiometry case the increased proton efflux via CAX was initially counteracted via the uncoupled passive efflux of chloride.

**Figure 6:**
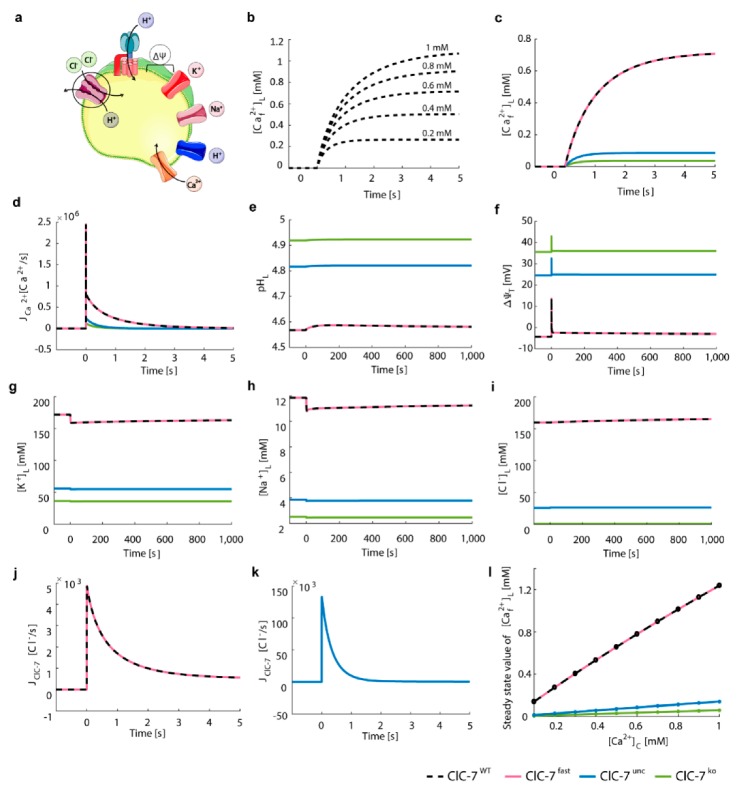
Cl-/H+ exchange supports channel-mediated lysosomal Ca^2+^ uptake independent of ClC-7 activation kinetics. (**a**) Schematic representation of the model with ClC-7 antiporters, V-ATPases, potassium, sodium, Ca^2+^ channels, and proton leak. The cartoon was created using Servier Medical Art templates (https://smart.servier.com), licensed under a Creative Commons License (https://creativecommons.org/licenses/by/3.0/). (**b**–**i**) The initial conditions were set to the steady-state values of Figure 4 (Table S3) and from t = 0 s, the lysosomal membrane was permeable to calcium ions (P_Ca_^2+^ = 5.7 × 10^−4^ cm/s) representing the opening of the uptake channel. (**b**) Luminal free Ca^2+^ concentration of ClC-7WT for five different values of cytosolic Ca^2+^ concentration ([Ca^2+^]_C_). (**c**–**k**) Depicted for the different ClC-7 scenarios during triggered Ca^2+^ uptake with a cytosolic Ca^2+^ concentration ([Ca^2+^]_C_) of 0.6 mM (ClC-7^WT^, dashed black line; ClC-7^fast^, red; ClC-7^unc^, blue; ClC-7^ko^, green) are (**c**) luminal free Ca^2+^ concentration, (**d**) Ca^2+^ flux, (**e**) luminal pH, (**f**) total membrane potential, luminal concentrations of (**g**) potassium, (**h**) sodium and (**i**) chloride ions, as well as the turnover rates of (**j**) ClC-7^WT^ and ClC-7^fast^, and (**k**) ClC-7^unc^. From t = 0 s, the lysosomal membrane was permeable to calcium ions (P_Ca_^2+^ = 5.7 × 10^−4^ cm/s) representing the opening of the uptake channel. (**l**) Steady state value of luminal free Ca^2+^ concentration for 10 different [Ca^2+^]_c_ values for the different ClC-7 scenarios.

We then simulated a channel-mediated ($N_{\mathrm{CAX}}=0$) Ca^2+^ uptake from the ER after Ca^2+^ release (steady-state conditions of Figure 4, Table S3) (Figure 6). Due to the reported close proximity of lysosomes to IP3 receptors of the ER a high extra-lysosomal concentration is expected that enables Ca^2+^ uptake by a low affinity Ca^2+^ transporter or channel [33].

As the Ca^2+^ concentration in the ER was reported to be between 100–800 μM [70], we tested different concentrations of free Ca^2+^ ranging from 0.2 mM to 1 mM. Our simulations showed a slightly higher luminal Ca^2+^ concentration as compared to the corresponding cytosolic values (Figure 6b). We then further evaluated further elements involved in lysosomal homeostasis for a fixed cytosolic Ca^2+^ concentration of 0.6 mM (Figure 6c–k). The Ca^2+^ permeability of the channel (P_Ca_^2+^ = 5.7 × 10^−4^ cm/s) was adjusted to mediate a 10-fold increase in the luminal free Ca^2+^ concentration within 1 s for ClC-7^WT^. Both the wild-type and fast ClC-7 lysosomes effectively accumulated Ca^2+^ (steady state [${\mathrm{Ca}}_{f}^{2+}{]}_{L}$ = 0.78 mM, Figure 6c). As the turnover rates of wild-type and fast ClC-7 were the same (Figure 6j), all simulated lysosomal elements displayed the same behaviour for these ClC-7 scenarios (Figure 6). Importantly, in the uncoupled and the knockout ClC-7 scenarios, the free lysosomal Ca^2+^ concentration remained drastically low (${\left[{\mathrm{Ca}}_{f}^{2+}\right]}_{L}$ = 0.09 and 0.04 mM, respectively, Figure 6c). This suggests an important role for Cl^−^/H^+^ exchange in the channel-mediated lysosomal Ca^2+^ uptake, which applied to all extra-lysosomal Ca^2+^ concentrations tested (Figure 6l and Figure S8).

### 3.5. Lysosomal Chloride Transport Affects Ca^2+^ Dynamics

**Figure 7:**
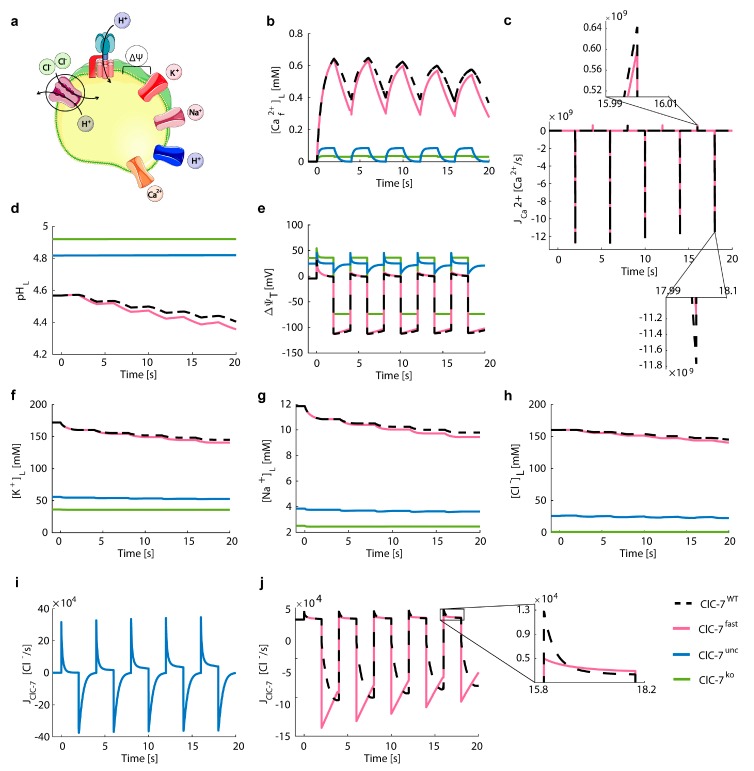
Ca^2+^ release accompanied exclusively by ClC-7 antiporter reveals differences between fast and WT scenarios. (**a**) Schematic representation of the model with ClC-7 antiporters, V-ATPases, potassium and sodium channels, proton leak, and Ca^2+^ release channel. The cartoon was created using Servier Medical Art templates (https://smart.servier.com), licensed under a Creative Commons License (https://creativecommons.org/licenses/by/3.0/). (**b**–**j**) Depicted for the different ClC-7 scenarios during subsequent Ca^2+^ uptake and release (ClC-7^WT^, dashed black line; ClC-7^fast^, red; ClC-7^unc^, blue; ClC-7^ko^, green) are (**b**) luminal free Ca^2+^ concentration, (**c**) Ca^2+^ flux with a zoom to the last simulated uptake (top) and release (bottom), (**d**) luminal pH, (**e**) total membrane potential, (**f**) luminal concentrations of potassium, (**g**) sodium, and (**h**) chloride ions, as well as the turnover rates of (**i**) ClC-7^WT^ and ClC-7^fast^, and (**j**) ClC-7^unc^. The initial conditions were set to the steady state values of Figure 4 (i.e., after Ca^2+^ release, Table S3). Ca^2+^ uptake and release was induced every 2 s by increasing and decreasing the cytosolic Ca^2+^ concentration, respectively. Ca^2+^ uptake was simulated considering all the elements shown in (**a**). In order to induce a change in the activity of the ClC-7 antiporter, we simulated Ca^2+^ release in the presence of only Ca^2+^ channel and ClC-7 antiporters.

Next, we investigated the impact of the different ClC-7 scenarios on subsequent cycles of lysosomal Ca^2+^ uptake and release. Starting from the steady-state values of Figure 4 (Table S3), we simulated the channel-mediated Ca^2+^ ($P_{{\mathrm{Ca}}^{2+}}$ = 5.7 × 10^−4^ cm/s, N_CAX_ = 0) uptake from the ER (mimicked by [Ca^2+^]_C_ = 0.6 mM) during 2 s, followed by Ca^2+^ release ($P_{{\mathrm{Ca}}^{2+}}$ = 0.58 cm/s, N_CAX_ = 0, [Ca^2+^]_C_ = 100 nM) during 2 s, as shown in Figure 7.

To create a scenario in which we expect differential Ca^2+^ dynamics between WT and fast ClC-7, we simulated Ca^2+^ release with only ClC-7 as possible counter-ion conductance (N_VATP_ = 0, P_H_^+^ = 0, P_K_^+^ = 0, P_Na_^+^ = 0) under which conditions we had observed a small difference between the fast and wild-type ClC-7 scenarios (Figure S3). We simulated a rapid change from Ca^2+^ release to Ca^2+^ uptake with the purpose of generating a fast change from large negative to small positive ClC-7 driving force that induces the deactivation of the ClC-7 antiporter. For this, the unique presence of the ClC-7 was not necessary, and therefore Ca^2+^ uptake was accompanied by a V-ATPase pump, proton leak, sodium, and potassium channels.

As seen in Figure 6, lysosomes with wild-type or fast Cl^−^/H^+^ exchangers accumulated Ca^2+^ to higher concentrations ([Ca^2+^]_L_ = 0.58 mM and 0.54 mM after the fourth release, respectively) than those with ClC-7^unc^ or ClC-7^ko^ ([Ca^2+^]_L_ = 0.08 mM and 0.03 mM after the fourth release, respectively, shown in Figure 7b). Indeed, ClC-7^WT^ and ClC-7^fast^ showed differences between their turnover rates (Figure 7j). Consequently, the counter-ion transport differed between these two scenarios, resulting in small differences in Ca^2+^ concentrations, which became larger with each cycle of Ca^2+^ uptake and release (Figure 7b). This was reflected in the differences in Ca^2+^ uptake and release (Figure 7c). In addition, in both cases, we observed continuous acidification which was slightly more pronounced for the fast ClC-7 scenario and which results from the contribution of proton influx via the Cl^−^/H^+^ exchanger as counter-ion for the Ca^2+^ release (Figure 7d). The Ca^2+^ uptake was counterbalanced by sodium and potassium efflux (Figure 7f,g). Due to the alternating Ca^2+^ uptake and release, the changes in the ClC-7 driving force led to different activity values and therefore to the activation and deactivation of ClC-7.

In sum, Figure 4b shows Ca^2+^ release from the steady state conditions of Figure 3 for all four ClC-7 scenarios. The Ca^2+^ release is predominantly driven by Ca^2+^ gradient, with the membrane potential and differential ionic concentration playing a minor role. Nevertheless, the results shown in Figure S3 (Ca^2+^ release without sodium and potassium channels) show that even when the Ca^2+^ gradient is the same in the different scenarios, the Ca^2+^ release would depend on the counter-transport availability, which in turn differ between the different ClC-7 scenarios. Moreover, in Figure 5 and Figure 6 we simulated the uptake of lysosomal Ca^2+^ via diverse potential mechanisms, which resulted in differential luminal Ca^2+^ concentrations in the different ClC-7 scenarios. Finally, the combination of Ca^2+^ uptake and subsequent release (Figure 7) is highly dependent on the membrane potential, ionic conditions, and therefore on the ClC-7 scenarios.

Altogether the results from our simulation point at differences in Ca^2+^ dynamics between scenarios where we have a Cl^−^/H^+^ exchanger (ClC-7^WT^ or ClC-7^fast^) and those without coupled transport (ClC-7^unc^ or ClC-7^ko^). For the ClC-7^unc^ and ClC-7^ko^ scenarios, a reduced in lysosomal chloride concentration has been reported for ClC-7^unc^ and ClC-7^ko^ cell lines [53] and for loss of the ClC-7 orthologue in *C. elegans* [45]. These results are in agreement with our prediction. Even though the wild-type and fast ClC-7 scenarios show similar kinetic behaviours, we could mimic an extreme situation where, in the absence of other charge compensating mechanisms, the exchanger plays a more prominent role in homeostasis and its different kinetics (wild-type vs. fast) lead to differences in output lysosomal ion concentrations.

## 4. Discussion

In this work, we developed and explored a new mathematical ODE-model for lysosomal ion homeostasis. Our model builds up on a previous ODE model for lysosomal acidification by Ishida et al. [62] and expands it by implementing the time-dependence of voltage gating of ClC-7-mediated Cl^−^/H^+^ exchange and most importantly by incorporating lysosomal Ca^2+^ uptake and release. It offers a mechanistic description of the impact of ClC-7 mutations on lysosomal Ca^2+^ release. We considered four different ClC-7 scenarios to investigate different levels of chloride transport disruption. Importantly, we aimed at including the minimal possible elements that could contribute to understanding the impact of chloride transport on Ca^2+^ dynamics. The different ClC-7 scenarios were simulated by changing the properties of the ClC-7 (either by considering instantaneous activation kinetics, by deleting completely the antiporter, or by simulating a channel-like antiporter). The uncoupled and knock-out scenarios were also described and simulated by Ishida et al. [62]. However, their impact on Ca^2+^ dynamics was not considered and to date it has not been experimentally validated.

With the developed model, we were able to investigate the effects of lysosomal acidification and Ca^2+^ dynamics depending on the presence, absence, and uncoupling and accelerating mutations of ClC-7 and to shed light on the patho-physiological impact of such mutations.

Like in previous mathematical models [53,62], we found that the exchange activity of ClC-7, which is absent in the ClC-7^unc^ and ClC-7^ko^ scenarios, leads to more efficient acidification. The role of ClC-7 in acidification is still a matter of debate [19,46], and the lysosomal pH has been found to be normally acidic in various cell types from ClC-7-deficient mice [39,48,53]. In our in silico simulations, cation conductance can support acidification to some extent, as also in a previous mathematical model by Ishida et al. [62] and as experimentally observed [39,53]. As previously suggested by another minimalistic mathematical model [53], the exchange activity of ClC-7 leads to an accumulation of Cl^−^ into lysosomes; and reduced lysosomal [Cl^−^] has indeed been observed upon ClC-7 depletion or uncoupling [45,53]. It is known that an acceleration of the ClC-7 voltage gating kinetics leads to osteopetrosis, as reported in humans and cattle, which is as severe as the phenotype caused by the loss of ClC-7 function. One possible scenario where we could envisage lysosomal membrane voltage jumps, for which the ClC-7 gating kinetics would matter, could be caused by sudden Ca^2+^ release. This assumption motivated us to introduce the transport of Ca^2+^ ions in the model.

Ca^2+^ plays major roles in various processes in lysosomal physiology, such as fusion and fission events and the regulation of mTORC1 signalling [4,27,28]. Several lysosomal Ca^2+^ channels are known with varying dependences on the transmembrane voltage and pH [28,34]. For simplicity, we opted for simulating the opening of a generic Ca^2+^ release channel by modulating Ca^2+^ permeability (*P_Ca_*^2+^); and additionally, we simulate a voltage dependent Ca^2+^ channel similar to TRPML1. Interestingly, in simulations of Ca^2+^ release for the same *P_Ca_*^2+^, starting at the same lysosomal Ca^2+^ concentrations, but for the different ClC-7 scenarios, the peak Ca^2+^ efflux, which is thought to be physiologically meaningful, was stronger for ClC-7^ko^ and ClC-7^unc^ due to their inside-positive potential.

Much less is known about the lysosomal Ca^2+^ uptake mechanism [28,34]. We tested two putative uptake mechanisms, (i) pH-dependent accumulation by an unknown H^+^/Ca^2+^ exchanger—we refer to this as CAX (as the underling protein(s) remains unknown, so does its coupling stoichiometry; therefore, we simulated various H^+^/Ca^2+^ stoichiometries) and (ii) uptake from the high Ca^2+^ concentration in the ER by a Ca^2+^ channel. Both transport mechanisms mediated efficient Ca^2+^ uptake.

Our simulations of Ca^2+^ uptake via a channel for a range of extra-lysosomal Ca^2+^ concentrations ([Ca^2+^]_c_) showed that efficient Ca^2+^ uptake by this mechanism [32] requires concentrations within the range of hundreds of μM. This would require tight coupling between the ER and lysosomes in agreement with published data on lysosomal sequestering of ER-released Ca^2+^ [71,72]. Such close interaction between lysosome and cluster of ER release channels (IP_3_ receptors) has been recently reported [33].

The Ca^2+^ uptake by CAX required a parallel Ca^2+^ leak, which could be provided by a release channel, for [Ca^2+^]_L_ to reach a steady-state level. For both simulated uptake mechanisms, lysosomes with a Cl^−^/H^+^ exchanger (in its wild-type or fast form), accumulated much more Ca^2+^ than those lysosomes with ClC-7^unc^ and ClC-7^ko^. The reduction in the steady-state values for [Ca^2+^]_L_ in lysosomes lacking Cl^−^/H^+^ exchange activity (ClC-7^unc^ and ClC-7^ko^) is less pronounced when Ca^2+^ is taken up by a CAX with a 2:1 or 3:1 H^+^/Ca^2+^ stoichiometry, as compared to a CAX with a 1:1 stoichiometry, or when uptake is mediated by a channel from high extra-lysosomal Ca^2+^. Given the lower [Ca^2+^]_L_ reached in ClC-7^unc^ and ClC-7^ko^, our observation of a faster Ca^2+^ release without the Cl^−^/H^+^ exchange activity from lysosomes with the same Ca^2+^ concentrations (see above) may not be physiologically relevant. In the consecutive cycles of Ca^2+^ release and uptake, which we simulated in consistence with the hypothesis that lysosomal Ca^2+^ refilling may be triggered directly by lysosomal Ca^2+^ release [34], the reduced lysosomal Ca^2+^ concentration also leads to a decrease in Ca^2+^ efflux. This is in agreement with the experimentally observed reduced Ca^2+^ release from lysosomes with lowered Cl^−^ concentrations [45].

Dysfunction of ClC-7 leads to osteopetrosis and lysosomal pathology [47,48]. This may be caused by an absence of the protein, subcellular mislocalization, uncoupling of Cl^−^ from H^+^ transport, or reduced ion transport capability [47,53,54,73], and surprisingly also by the acceleration of the normally relatively slow voltage-gating of ClC-7 [54,59]. An existing mathematical model of resorption lacuna acidification developed by Marcoline et al. [65], refers to the exocytosis of lysosomes as a Ca^2+^-mediated process, yet the authors did not include Ca^2+^ in the model, nor explicitly considered the lysosomal compartment. Instead, the authors activate or de-activate V-ATPases and ClC-7 antiporters mimicking the fusion of the lysosomes with the ruffled border, which then results in the acidification of the pit and do not consider different osteopetrosis-associated ClC-7 mutations, unlike in our study.

To investigate how changes in ClC-7 (de)activation kinetics impinge on lysosomal ion homeostasis and dynamics, we implemented the time dependence of voltage-dependent ClC-7 (de)activation in our model. With this approach, we could in silico recapitulate electrophysiological measurements of the wild-type ClC-7 [54,62,67,74] and of its acceleration in disease-causing mutations [54,59,60]. As expected, our simulations of lysosomal acidification were not affected by changes in the time-dependence of ClC-7, since the steady-state values for the ClC-7 driving force did not induce changes in the activity of the antiporter. Lysosomal Ca^2+^ uptake, both by CAX and by a Ca^2+^ channel, was unaffected by changes in the time-dependence of voltage-gated ClC-7 activation. Importantly, acceleration of the (de)activation kinetics did not affect Ca^2+^ release (unless we simulated an unlikely situation without Na^+^ and K^+^ channels) when it was simulated as a single event. When we simulated release and uptake cycles, consistent with the hypothesis that lysosomal Ca^2+^ refilling can be directly triggered by lysosomal Ca^2+^ release [34], we generated rapid changes between a large negative and a small positive ClC-7 driving force that induced the (de)activation of the ClC-7 antiporter. Under these conditions, we could indeed observe differences between the behaviours of wild-type and fast ClC-7 that resulted in differences in Ca^2+^ concentrations and peak Ca^2+^ release, which was stronger in the wild-type scenario (Figure 7). Lysosomal Ca^2+^ release may be a key factor in the pathogenesis of ClC-7-associated osteopetrosis, since osteoclasts lacking ClC-7 (ClC-7^ko^) or expressing an uncoupled ClC-7 mutation (ClC-7^unc^) present underdeveloped ruffled borders [47,53], likely due to decreased lysosomal exocytosis, a process involving lysosomal Ca^2+^ release. Yet, the small differences generated by the acceleration of our in silico ClC-7^fast^, which mimics an extreme case of acceleration, compared to that observed for osteopetrosis-causing ClC-7 mutants [54,59,60], are unlikely to cause the pathology. Thus, ClC-7 acceleration exerts its detrimental effect likely not only via its effect on this simple ion equilibria but likely supported by other, more complex interconnections and regulatory pathways, which may include temporal regulation via the circadian system [75]. In fact, several of the genes which are mutated in lysosomal storage pathologies, code for circadian transcripts and circadian variation was reported in lysosomal enzymes, implying a 24-h rhythmicity in lysosomal functioning [76]. This complexity is further seen from our in silico observation of mild acidification during Ca^2+^ release (due to H^+^ influx serving as part of the counterion transport) whereas triggered release of Ca^2+^ by NAADP was found to be paralleled by a lysosomal alkalinisation [77]. In addition, the reported heterogeneity in lysosomal population with respect to localization and possibly composition of ion transporters (ClC-7 might not be present in all lysosomes) may add to the complexity of the system and the variability of experimental observations [13,14,78]. The reasons for the large discrepancies between the reported values for lysosomal-related parameters may be manifold, including different experimental conditions, different cell lines under study, different experimental techniques, etc. Our model will be available to the scientific community at BioModels and can be used to simulate numerous scenarios applicable to the different experimental models and conditions.

Our model enables a detailed analysis of the potential role of ClC-7 in the dynamics of Ca^2+^ and in the overall lysosomal ion homeostasis. It would be interesting, in future work, to couple this model with a mathematical model of the circadian clock [79] and introduce circadian regulation into the system. In addition, given the important role of lysosomes [80,81,82] and the circadian clock [83,84,85,86] in cancer, it would be relevant to further investigate the possible interplay between the circadian clock and lysosomes in a cancer context.

Moreover, a detailed experimental analysis will be possible with the development of novel ion and voltage sensors [23,44,78,87] and will allow for the testing and validations from predictions from our model, as presented in this work. Altogether, such developments will allow testing the validity of our predictions regarding the link of ClC-7 and Ca^2+^ uptake/release and to obtain a more mechanistic picture of lysosomal ion homeostasis.

## Supplementary Materials

The following are available online at https://www.mdpi.com/2073-4409/8/10/1263/s1, Supplementary Materials Section S1. Mathematical model of lysosomal ion homeostasis; Section S2. Conditions for the (de)activation of the ClC-7 antiporter and differentiation between fast and WT scenarios; Section S3. Steady-state values of three simulations; Section S4. Sensitivity analysis.
