# Supplementary material for: A Mathematical Model of Lysosomal Ion Homeostasis Points to Differential Effects of Cl− Transport in Ca2+ Dynamics
Source: Cells. 2019 Oct 16;8(10):1263. doi: 10.3390/cells8101263 (PMC6848924; doi:10.3390/cells8101263)
Supplement: Supplementary file 1 [file cells-08-01263-s001.pdf]

Supplementary Material

A mathematical model of lysosomal ion homeostasis points to differential effects of Cl^- transport in Ca^{2+} dynamics

Rosario Astaburuaga [1,2], Orlando Daniel Quintanar Haro [1,3], Tobias Stauber [3,4,*,π], Angela Relógio [1,2,*,π]

[1] Institute for Theoretical Biology (ITB), Charité-Universitätsmedizin Berlin, Corporate Member of the Freie Universität Berlin, Humboldt-Universität zu Berlin, and Berlin Institute of Health, 10115 Berlin, Germany
[2] Medical Department of Hematology, Oncology and Tumor Immunology, Molekulares Krebsforschungzentrum (MKFZ), Charité-Universitätsmedizin Berlin, Corporate Member of the Freie Universität Berlin, Humboldt-Universität zu Berlin, and Berlin Institute of Health, 13353 Berlin, Germany
[3] Freie Universität Berlin, Institute of Chemistry and Biochemistry, 14195 Berlin, Germany
[4] Department of Human Medicine, Medical School Hamburg, 20457 Hamburg, Germany
[π] These authors contributed equally for the work
* Correspondence: angela.relogio@charite.de (AR), tobias.stauber@fu-berlin.de (TS)

Contents

1. Mathematical model of lysosomal ion homeostasis
2. Conditions for the (de)activation of the ClC-7 antiporter and differentiation between fast and WT scenarios
3. Steady-state values of three simulations
4. Sensitivity analysis
5. Supplementary Figures

1. Mathematical model of lysosomal ion homeostasis

The equations written in purple are newly developed, the other elements were retrieved from a previous mathematical model as indicated above. All parameters and variables are listed in **Tables S1 and S2**, respectively.

Membrane potential

Our model includes a description of the membrane potential [1, 2], which depends on the concentration of ions within the lysosomal lumen:

$$\Delta\psi = \frac{F \cdot V}{C \cdot S}([H^+]_L + [K^+]_L + [Na^+]_L - [Cl^-]_L + \mathbf{2[Ca^{2+}]_L} - B) \quad \text{(S1)}$$

By convention, the membrane potential ($\Delta\psi$) is negative if the number of anions inside the lysosome (luminal) is higher relative to the outside (cytosol). The total membrane potential ($\Delta\psi_T$) accounts for the intrinsic charge on the outer (ψ_{out}) and inner leaflets (ψ_{in}) of the lysosomal membrane[2]:

$$\Delta\psi_T = \Delta\psi + (\psi_{\text{out}} - \psi_{\text{in}}) \quad \text{(S2)}$$

For each simulation, the initial value for the concentration of Donnan particles (B) was adjusted to set a null initial total membrane potential ($\Delta\psi_T = 0$).

Modified ion concentrations

To account for the effects of leaflet potential, the cytoplasmic and luminal concentrations were modified by a Boltzmann factor, leading to cytoplasmic and luminal surface concentrations:

$$pH_i = pH_L + \frac{\psi_{in} \cdot F}{2.3 \cdot R \cdot T} \quad \text{(S3)}$$

$$pH_e = pH_C + \frac{\psi_{out} \cdot F}{2.3 \cdot R \cdot T} \quad \text{(S4)}$$

$$[Cl^-]_i = [Cl^-]_L \cdot \exp\left(\frac{\psi_{\text{in}} \cdot F}{R \cdot T}\right) \quad \text{(S5)}$$

$$[Cl^{-}]_e = [Cl^{-}]_C \cdot \exp\left(\frac{\psi_{\text{out}} \cdot F}{R \cdot T}\right) \tag{S6}$$

$$[K^{+}]_i = [K^{+}]_L \cdot \exp\left(\frac{-\psi_{\text{in}} \cdot F}{R \cdot T}\right) \tag{S7}$$

$$[K^{+}]_e = [K^{+}]_C \cdot \exp\left(\frac{-\psi_{\text{out}} \cdot F}{R \cdot T}\right) \tag{S8}$$

$$[Na^{+}]_i = [Na^{+}]_L \cdot \exp\left(\frac{-\psi_{\text{in}} \cdot F}{R \cdot T}\right) \tag{S9}$$

$$[Na^{+}]_e = [Na^{+}]_C \cdot \exp\left(\frac{-\psi_{\text{out}} \cdot F}{R \cdot T}\right) \tag{S10}$$

$$[Ca_f^{2+}]_i = [Ca_f^{2+}]_L \cdot \exp\left(\frac{-2\psi_{\text{in}} \cdot F}{R \cdot T}\right) \tag{S11}$$

$$[Ca_f^{2+}]_e = [Ca^{2+}]_C \cdot \exp\left(\frac{-2\psi_{\text{out}} \cdot F}{R \cdot T}\right) \tag{S12}$$

$$[Ca_T^{2+}]_i = [Ca_T^{2+}]_L \cdot \exp\left(\frac{-2\psi_{\text{in}} \cdot F}{R \cdot T}\right) \tag{S13}$$

$$[Ca_T^{2+}]_e = [Ca^{2+}]_C \cdot \exp\left(\frac{-2\psi_{\text{out}} \cdot F}{R \cdot T}\right) \tag{S14}$$

The subscripts *i* and *e* indicate internal (luminal) and external (cytosolic), respectively. *R* is the gas constant and *F* is the Faraday's constant. At room temperature (T = 25 ^{0}C), R·T/F = 25.69 mV. This value was used in all the simulations.

Number of ions

The number of luminal ions is calculated based on the concentration, the lysosome volume (*V*), and the Avogadro's number (N_A):

$$NH^{+} = [H^{+}]_L \cdot V \cdot N_A \tag{S15}$$

$$NCl^{-} = [Cl^{-}]_L \cdot V \cdot N_A \tag{S16}$$

$$NK^{+} = [K^{+}]_{L} \cdot V \cdot N_{A} \tag{S17}$$

$$NNa^{+} = [Na^{+}]_{L} \cdot V \cdot N_{A} \tag{S18}$$

$$NCa_{T}^{2+} = [Ca_{T}^{2+}]_{L} \cdot V \cdot N_{A} \tag{S19}$$

$$NCa_{f}^{2+} = NCa_{T}^{2+} \cdot V \cdot N_{A} \cdot r_{Ca^{2+}} \tag{S20}$$

$r_{Ca^{2+}}$ is the ratio of total to free calcium. As the cytoplasmic values of these ions change very little during acidification, the cytosolic concentrations were considered to be constant.

Rate of change of the ions within the lysosomal lumen

The rate of change of lysosomal pH (pH_L) is determined by the change of luminal proton concentration and the buffering capacity of the lumen (β):

$$\frac{dpH_{L}}{dt} = -\frac{1}{\beta}\left(\frac{dNH^{+}}{dt} \cdot \frac{1}{V \cdot N_{A}}\right) \tag{S21}$$

In the equation above, the change in the number of luminal protons is determined by

$$\frac{dNH^{+}}{dt} = \begin{cases} J_{VATP} - n_{H^{+}}^{ClC-7} \cdot J_{ClC-7^{WT}} - n_{H^{+}}^{CAX} \cdot J_{CAX} + J_{H^{+}} & \text{, for ClC-7}^{\text{WT}} \\ J_{VATP} - n_{H^{+}}^{ClC-7} \cdot J_{ClC-7^{fast}} - n_{H^{+}}^{CAX} \cdot J_{CAX} + J_{H^{+}} & \text{, for ClC-7}^{\text{fast}} \\ J_{VATP} - n_{H^{+}}^{CAX} \cdot J_{CAX} + J_{H^{+}} & \text{, for ClC-7}^{\text{unc}} \text{ and ClC-7}^{\text{ko}} \end{cases} \tag{S22}$$

J_{VATP} is the proton pumping rate of the V-ATPase pump (positive for proton influx), $J_{ClC-7^{WT}}$ and $J_{ClC-7^{fast}}$ are the turnover rates (positive for proton efflux) for ClC-7^{WT} and ClC-7^{fast}, respectively. J_{CAX} is the turnover rate of a calcium/proton exchanger CAX (positive for proton efflux), and $J_{H^{+}}$ is the passive, non-voltage activated proton flux through channel (positive for proton influx). The stoichiometries of ClC-7 and CAX for proton counter-transport are specified by $n_{H^{+}}^{ClC-7}$ and $n_{H^{+}}^{CAX}$, respectively.

The rate of change of luminal chloride ions is described by

$$\frac{dNCl^-}{dt} = \begin{cases} n_{Cl^-}^{ClC-7} \cdot J_{ClC-7^{WT}} & \text{, for ClC-7}^{\text{WT}} \\ n_{Cl^-}^{ClC-7} \cdot J_{ClC-7^{fast}} & \text{, for ClC-7}^{\text{fast}} \\ J_{ClC-7^{unc}} & \text{, for ClC-7}^{\text{unc}} \\ J_{ClC-7^{ko}} & \text{, for ClC-7}^{\text{ko}} \end{cases} \quad \text{(S23)}$$

$n_{Cl^-}^{ClC-7}$ is the ClC-7 stoichiometry for chloride, and $J_{ClC\text{-}7^{WT}}$, $J_{ClC\text{-}7^{fast}}$, $J_{ClC\text{-}7^{unc}}$, *and* $J_{ClC\text{-}7^{ko}}$ are the scenario-specific ClC-7 turnover rates (positive for chloride influx).

The number of potassium ions within the lysosomal lumen varies due to their passive flow across the lysosomal membrane:

$$\frac{dNK^+}{dt} = J_{K^+} \quad \text{(S24)}$$

J_{K^+} is the turnover rate of the non-voltage activated potassium channel (positive for potassium influx).

Similarly, the rate of change in luminal sodium ions is determined by

$$\frac{dNNa^+}{dt} = J_{Na^+} \quad \text{(S25)}$$

J_{Na^+} is the turnover rate of the sodium channel (positive for sodium influx).

The change in total calcium ions is described as

$$\frac{dNCa_T^{2+}}{dt} = n_{Ca^{2+}}^{CAX} \cdot J_{CAX} + J_{Ca^{2+}} + J_{TRPML1} \quad \text{(S26)}$$

J_{CAX} is the turnover rate of CAX (positive for calcium influx), $n_{Ca^{2+}}^{CAX}$ is the CAX stoichiometry for calcium, $J_{Ca^{2+}}$ is the passive flow through a calcium channel (positive for calcium influx), and J_{TRPML1} is the voltage- and pH- dependent flow through TRPML1 channel (positive for calcium influx).

Due to calcium buffering, the rate in luminal free calcium within the lumen is determined by the variation in total luminal calcium and the ratio ($r_{Ca^{2+}}$) of total to free calcium:

$$\frac{dNCa_f^{2+}}{dt} = \frac{dNCa_T^{2+}}{dt} \cdot r_{Ca^{2+}} \quad \text{(S27)}$$

Turnover rates

The pumping rate of the V-ATPase (J_{VATP}) is given by a detailed mechanochemical model, which was calibrated against experimental data for current voltage [3]. J_{VATP} which depends on the luminal pH (pH_L) and on the membrane potential ($\Delta\psi$):

$$J_{VATP} = N_{VATP} \cdot J_{VATP1}\,(pH_L, \Delta\psi) \tag{S28}$$

N_{VATP} is the number of V-ATPase pumps located in the lysosomal membrane, and J_{VATP1} is the proton pumping rate of a single V-ATPase under different membrane potentials and pH gradients. The proton pumping profile was generated using the model of Grabe *et al.*[3] with values for the membrane potential ($\Delta\psi$) varying from -200 to 500 mV in increments of 1 mV, and the luminal pH (pH_L) values to vary from 1 to 14 in increments of 0.1. A linear interpolation is used to obtain the corresponding pumping rate, if the input luminal pH and membrane potentials were not the same values for the pumping profile generation. The model assigns boundary values of pumping rate if the input value for the membrane potential is lower than -200 mV or higher than 500 mV.

The equation for the ClC-7 turnover rate formulated by Ishida *et al.*[2] is time-independent and represents an instantaneous (de)activation of the antiporter. Therefore, we used the same mathematical description to represent the ClC-7^{fast} turnover rate, but modified the equation to have an explicit term for the ClC-7 activity (A). Thus, the ClC-7^{fast} turnover rate is given by

$$\boldsymbol{J_{ClC-7^{fast}} = N_{ClC-7} \cdot A \cdot \Delta\mu_{ClC-7}} \tag{S29}$$

N_{ClC-7} is the number of ClC-7 antiporters, $\Delta\mu_{ClC-7}$ is the turnover driving force[2]:

$$\Delta\mu_{ClC-7} = (n_{H^+}^{ClC-7} + n_{Cl^-}^{ClC-7}) \cdot \Delta\psi + \frac{R \cdot T}{F}\left(2.3 \cdot n_{H^+}^{ClC-7} \cdot (pH_e - pH_i) + n_{Cl^-}^{ClC-7} \cdot \ln\frac{[Cl^-]_e}{[Cl^-]_i}\right) \tag{S30}$$

and A is the activity

$$\boldsymbol{A = 0.3 \cdot x + 1.5 \cdot 10^{-5} \cdot (1 - x) \cdot \Delta\mu^2_{ClC-7}} \quad \text{(S31)}$$

The switching function x[2] varies between zero at negative membrane potentials ($\Delta\psi$) and 1 at positive $\Delta\psi$:

$$x = 0.5 + 0.5 \cdot \tanh\left(\frac{\Delta\mu_{ClC-7} + 250}{75}\right) \quad \text{(S32)}$$

In the equations above, $n_{Cl^-}^{ClC-7}$ and $n_{H^+}^{ClC-7}$ are the ClC-7 stoichiometries for chloride and protons, respectively. The subscripts i and e represent the modified luminal and cytosolic quantities, respectively (**Equations S5 and S6**).

We described the ClC-7^{WT} turnover rate as a function of the effective activity A_{eff} :

$$\boldsymbol{J_{ClC-7^{WT}} = N_{ClC-7} \cdot A_{eff} \cdot \Delta\mu_{ClC-7}} \quad \text{(S33)}$$

N_{CLC-7} is the number of ClC-7 antiporters, $\Delta\mu_{ClC-7}$ is the driving force (**Equation S30**), and A_{eff} is defined as:

$$\boldsymbol{\frac{dA_{eff}}{dt} = \frac{1}{\tau} \cdot (A - A_{eff})} \quad \text{(S34)}$$

with A the ClC-7 activity (**Equation S31**). In the equation above, if A is higher (lower) than A_{eff}, then A_{eff} increases (decreases) according to the activation (deactivation) time $\tau = \tau_{act}$ ($\tau = \tau_{deact}$) until it reaches the activity A. We define "activity" as a variable related to an open probability, which determines the (de)activation kinetics of the ClC-7 turnover rate. The effective activity A_{eff} reaches the activity A after a certain amount of time determined by the activation (τ_{act}) or deactivation time (τ_{deact}). For simplicity, we considered the deactivation time τ_{deact} to be proportional to the activation time τ_{act}:

$$\boldsymbol{\tau_{deact} = \tau_{act} \cdot r_\tau} \quad \text{(S35)}$$

With r_τ the deactivation to activation ratio.

The uncoupled transport of chloride was simulated as a passive chloride flux through a "channel-like" ClC-7 antiporter. Therefore, we describe the ClC-7^{unc} turnover rate as

$$J_{ClC-7^{unc}} = P_{Cl^-} \cdot S \cdot \frac{U}{1-e^{-U}} \cdot ([Cl^-]_e - [Cl^-]_i \cdot e^{-U}) \cdot \frac{N_A}{10^3} \tag{S36}$$

P_{Cl^-} is the permeability per unit area for chloride ions, S is the lysosome surface area, N_A is the Avogadro's number, $U = (\Delta\psi \cdot F)/(R \cdot T)$ is the reduced membrane potential, and $[Cl^-]_e$, $[Cl^-]_i$ are the modified cytosolic and luminal chloride concentration, respectively.

The turnover rate for the ClC-7 knockout ($J_{ClC-7^{ko}}$) was calculated with **Equation S33** setting $N_{ClC-7} = 0$.

The turnover rate for CAX is described by

$$\boldsymbol{J_{CAX} = N_{CAX} \cdot \Delta\mu_{CAX}} \tag{S37}$$

with the driving force $\Delta\mu_{CAX}$ defined as

$$\boldsymbol{\Delta\mu_{CAX} = (n_{H^+}^{CAX} - 2 \cdot n_{Ca^{2+}}^{CAX}) \cdot \Delta\psi + \frac{RT}{F}\left(2.3 \cdot n_{H^+}^{CAX} \cdot (pH_e - pH_i) + \frac{n_{Ca^{2+}}^{CAX}}{2} \cdot \ln\frac{[Ca_f^{2+}]_i}{[Ca_f^{2+}]_e}\right)} \tag{S38}$$

The previous equation was created based on the driving force for ClC-7 (**Equation S30**).

Passive, non-voltage activated ion fluxes

The proton flux through the channel is described by[2]

$$J_{H^+} = P_{H^+} \cdot S \cdot \frac{U}{1-e^{-U}} \cdot (10^{-pH_e} \cdot e^{-U} - 10^{-pH_i}) \cdot \frac{N_A}{10^3} \tag{S39}$$

With P_{H^+} the permeability per unit area for protons, and $U = (\Delta\psi \cdot F)/(R \cdot T)$ the reduced membrane potential. Similarly, the passive flows for potassium, sodium and calcium ions through their corresponding channels are described by **Equation S40, S41,** and **S42**, respectively.

$$J_{K^+} = P_{K^+} \cdot S \cdot \frac{U}{1-e^{-U}} \cdot ([K^+]_e \cdot e^{-U} - [K^+]_i) \cdot \frac{N_A}{10^3} \tag{S40}$$

$$J_{Na^+} = P_{Na^+} \cdot S \cdot \frac{U}{1-e^{-U}} \cdot ([Na^+]_e \cdot e^{-U} - [Na^+]_i) \cdot \frac{N_A}{10^3} \tag{S41}$$

$$\boldsymbol{J_{Ca^{2+}} = P_{Ca^{2+}} \cdot S \cdot \frac{2U}{1-e^{-2U}} \cdot \left([Ca_f^{2+}]_e \cdot e^{-2U} - [Ca_f^{2+}]_i\right) \cdot \frac{N_A}{10^3}} \tag{S42}$$

Voltage and pH activated calcium flux

The calcium flux through the TRPML1-like channel was described as

$$J_{TRPML1} = P_{TRPML1} \cdot S \cdot \frac{2U}{1 - e^{-2U}} \cdot \left(\left[Ca_f^{2+}\right]_e \cdot e^{-2U} - \left[Ca_f^{2+}\right]_i\right) \cdot \frac{N_A}{10^3} \quad \text{(S43)}$$

where the permeability (P_{TRPML1}) depends on the luminal pH and on the membrane potential[4, 5]:

$$P_{TRPML1} = 3.88 \cdot 10^{-9}\left(y|\Delta\Psi| + (1 - y)\frac{|\Delta\Psi + 40|^3}{{pH_L}^{2.2}}\right) \quad \text{(S44)}$$

$$y = 0.5 - 0.5\tanh(\Delta\Psi + 40) \quad \text{(S45)}$$

The function y goes from 1 at $\Delta\psi$ < -40 mV, to 0 at $\Delta\psi$ ≥ -40 mV. Hence, for membrane potentials lower than -40 mV, the permeability of the TRPML1 channel is directly proportional to the membrane potential and does not depend on the luminal pH [4]. For membrane potentials higher than -40 mV, the P_{TRPML1} is directly proportional to the membrane potential to the power of three, and inversely proportional to the luminal pH, as revealed on the patch-clamp experiments of Dong *et al.* [4]. The constants 3.88x10^{-9} and 2.2 were set such as the ratio of P_{TRPML1} at $pH_L = 4.6, \Delta\Psi = 80\ mV$ versus $pH_L = 7.4, \Delta\Psi = 80\ mV$ was close to 2.82, and the ratio at $pH_L = 6.0, \Delta\Psi = 80\ mV$ versus $pH_L = 7.4, \Delta\Psi = 80\ mV$ was close to 1.35, as retrieved from the patch-clamp experiments of Dong *et al.* [4].

Table S1. Model parameters.

Description	Units	Symbol	Value	Reference
1) Initial cytosolic pH		pH_C	7.2	[6, 7]
2) Initial cytosolic sodium concentration	M	$[Na^+]_C$	0.01	[8]
3) Initial cytosolic potassium concentration	M	$[K^+]_C$	0.145	[8]
4) Initial cytosolic chloride concentration	M	$[Cl^-]_C$	0.01	[8]
5) Initial cytosolic calcium concentration	M	$[Ca^{2+}]_C$	10^{-7} [a]	[9]
6) Initial luminal pH		$pH_{L,0}$	6	[10]
7) Initial luminal sodium concentration	M	$[Na^+]_{L,0}$	0.02	[10]
8) Initial luminal potassium concentration	M	$[K^+]_{L,0}$	0.05	[10]
9) Initial luminal chloride concentration	M	$[Cl^-]_{L,0}$	0.001	[10]
10) Initial luminal total calcium concentration	M	$[Ca_T^{2+}]_{L,0}$	6×10^{-3}	[11]
11) Free to total calcium ratio		$r_{Ca^{2+}}$	0.1	[11]
12) Proton permeability	cm/s	P_{H^+}	6×10^{-5}	[2]
13) Sodium permeability	cm/s	P_{Na^+}	9.6×10^{-7} [b]	[12]
14) Potassium permeability	cm/s	P_{K^+}	7.1×10^{-7} [b]	[12]
15) Chloride permeability	cm/s	P_{Cl^-}	1.2×10^{-5} [c]	[12]
16) Calcium permeability	cm/s	$P_{Ca^{2+}}$	1.49×10^{-7} [d]	
17) Number of V-ATPase pump		N_{VATP}	300	[2]
18) Number of ClC-7 antiporters		$N_{ClC\text{-}7}$	300 [e]	[2]
19) Number of CAX antiporters		N_{CAX}	10 [f]	
20) Chloride stoichiometry of ClC-7		$n_{Cl^-}^{ClC7}$	2	[13]

21) Proton stoichiometry of ClC-7		$n_{H^+}^{ClC7}$	1	[13]
22) Calcium stoichiometry of CAX		$n_{Ca^{2+}}^{CAX}$	1	[14]
23) Proton stoichiometry of CAX		$n_{H^+}^{CAX}$	3 [f]	[14]
24) Activation time of ClC-7	s	τ_{act}	1 [g]	[13]
25) Deactivation to activation ratio		r_τ	0.25 [h]	[15]
26) Initial effective activity		$A_{eff,0}$	0.3 [i]	
27) Bilayer capacitance	F/cm^2	C	10^{-6}	[16]
28) Lysosome volume	L	V	1.65×10^{-16}	[2]
29) Lysosome surface area	cm^2	S	1.45×10^{-8}	[2]
30) Proton buffering capacity	M/pH	β	0.04	[2]
31) Donnan particles concentration	M	B	0.081 [j]	
32) Outside leaflet potential	mV	ψ_{out}	-50	[2]
33) Inside leaflet potential	mV	ψ_{in}	0	[2]

[a] This parameter was set to 0.6 mM to mimic the channel-mediated calcium uptake from the ER [17] for the simulation of **Figures 6 and 7** in the main text.

[b] This parameter was set to 0 for the simulation of **Figure S3**.

[c] This value was used for the simulation of the uncoupled transport of chloride through ("channel-like") ClC-7 (ClC-7^{unc}). This parameter was set to zero for the simulation of ClC-7^{ko}, ClC-7^{WT}, and ClC-7^{fast}.

[d] This value was used to provide steady state calcium concentrations as a complementary calcium leak via channel for calcium uptake via 10 CAXs with 3:1 stoichiometry (see **Figure S5**). This parameter was further adjusted in simulations for **Figures 4-7** and for **Figures S3-S8.** The used values were specified in the corresponding figure legend.

[e] This value was used for the simulation of ClC-7^{WT} and ClC-7^{fast}. This parameter was set to zero for the simulation of ClC-7^{unc}.

[f] These values were used for the simulation of **Figure S5**. These parameters were varied for the simulations of **Figures S4-S7**. N_{CAX} was set to zero for the simulations of **Figures 3**,**4**,**6**,**7**, and Figures S2 and S3.

[g] The activation time varies with the pH. We used this constant activation time found for acidic oocytes[13] for the simulation of WT scenario. This parameter was varied in **Figure 2** in the main text.

[h] The deactivation time was found to be around a quarter of the activation time [15].

[i] Value equal to the activity A that corresponds to an initial null driving force (see **Equations S29-S31**).

[j] This value was kept constant for all simulations performed in this study and was adjusted to set the initial $\Delta\psi_T$ to 0 mV.

Table S2. Model variables.

Description		Units	Symbol
1)	Luminal pH		pH_L
2)	Membrane potential	mV	$\Delta\psi$
3)	Total membrane potential	mV	$\Delta\psi_T$
4)	Number of protons		NH^+
5)	Luminal proton concentration	M	$[H^+]_L$
6)	Number of chloride ions		NCl^-
7)	Luminal chloride concentration	M	$[Cl^-]_L$
8)	Number of potassium ions		NK^+
9)	Luminal potassium concentration	M	$[K^+]_L$
10)	Number of sodium ions		NNa^+
11)	Luminal sodium concentration	M	$[Na^+]_L$
12)	Number of total calcium ions		NCa_T^{2+}
13)	Luminal total calcium concentration	M	$[Ca_T^{2+}]_L$
14)	Number of free calcium ions		NCa_f^{2+}
15)	Luminal free calcium concentration	M	$[Ca_f^{2+}]_L$
16)	Activity		A
17)	Effective activity		A_{eff}
18)	Switching function for ClC-7		x
19)	Modified luminal pH		pH_i
20)	Modified luminal proton concentration	M	$[H^+]_i$

21)	Modified luminal chloride concentration	M	$[Cl^-]_i$
22)	Modified luminal potassium concentration	M	$[K^+]_i$
23)	Modified luminal sodium concentration	M	$[Na^+]_i$
24)	Modified luminal total concentration	M	$[Ca_T^{2+}]_i$
25)	Modified luminal free calcium concentration	M	$[Ca_f^{2+}]_i$
26)	Total turnover rate of V-ATPase	H^+/s	J_{VATP}
27)	Driving force of ClC-7	mV	$\Delta\mu_{ClC-7}$
28)	Total turnover rate of ClC-7	Cl^-/s	$J_{ClC-7^{WT}}, J_{ClC-7^{fast}},$ $J_{ClC-7^{unc}}, J_{ClC-7^{ko}}$
29)	Driving force of CAX	mV	$\Delta\mu_{CAX}$
30)	Total turnover rate of CAX	Ca^{2+}/s	J_{CAX}
31)	Rate of proton channel	H^+/s	J_{H^+}
32)	Rate of potassium channel	K^+/s	J_{K^+}
33)	Rate of sodium channel	Na^+/s	J_{Na^+}
34)	Rate of calcium channel	Ca^{2+}/s	$J_{Ca^{2+}}$
35)	Rate of TRPML1-like channel	Ca^{2+}/s	J_{TRPML1}
36)	Switching function for TRPML1-like channel		y

2. Conditions for the (de)activation of the ClC-7 antiporter and differentiation between fast and WT scenarios

This section provides a detailed description of the conditions needed for the (de)activation of the ClC-7 antiporter, according to our mathematical model. We provide an explanation for the equivalent behaviour of the ClC-7^{fast} and ClC-7^{WT} antiporter as depicted in **Figures 3**, **4**, and **6**, and for the differential behaviour between these two scenarios observed in **Figure 7**, and **Figure S3**.

The direction of the chloride current through the ClC-7 antiporter is determined by the sign of the driving force ($\Delta\mu_{ClC-7}$, **Equation S30**), and consequently by the sign of the turnover rate (J_{ClC-7}, **Equations S29 and S33**). Therefore, positive (negative) values of driving force lead to chloride going into (out of) the lysosome. The outwardly rectifying behaviour of the ClC-7 antiporter is represented by the activity A (**Equation S31**), which depends on the driving force ($\Delta\mu_{ClC-7}$) and on the switching function (x, **Equation S32**). The slow voltage-gated (de)activation of the ClC-7 antiporter is described by an ODE representing the temporal evolution of the effective activity A_{eff} (**Equation S34**), i.e., the activity that the ClC-7 antiporter can actually achieve at a certain time. The turnover rate of the WT ClC-7 ($J_{ClC\text{-}7^{WT}}$), as a strong outwardly rectifier and slowly voltage-gated antiporter, is calculated based on the effective activity A_{eff} (**Equation S33**).

As the fast scenario mimics a ClC-7 antiporter with instantaneous (de)activation kinetics, the activation A is instantaneously achieved, and therefore is directly used for the computation of the turnover rate of the fast ClC-7 ($J_{ClC\text{-}7^{fast}}$, **Equation S29**).

The relationship between the activity A and the driving force $\Delta\mu_{ClC\text{-}7}$ was calculated from **Equation S31 and S32** by varying the value of the driving force from -500 mV to 500 mV (**Figure S1**). The value of the activity is set to 0.3 for values higher than -155 mV. As the activity does not change for driving forces between -155 mV and +∞, an activation or deactivation of the ClC-7 will not occur in this range. Under these conditions, ClC-7^{fast} and ClC-7^{WT} display the same turnover rate ($J_{ClC\text{-}7}$), *i.e.* the same behaviour. The initial effective activity was set to $A_{eff,0}$ = 0.3 (**Table S1**), equivalent to the value of activity A for an initial null driving force.

Hence, differences between ClC-7^{fast} and ClC-7^{WT} are observed only for driving forces lower than -155 mV, *i.e.* in a domain in which the value of the activity is not constant, inducing a change in the effective activity from its initial value. In this scenario, a slow (for ClC-7^{WT}) or instantaneous (for ClC-7^{fast}) activation would be induced.

In the simulations of **Figures 3, 4, and 6** in the main text, the driving forces of ClC-7^{fast} and ClC-7^{WT} antiporters did not reach values lower than -155 mV (**Figure S1b, c, and d,** respectively). Therefore, the value of the activity was constant ($A = A_{eff} = 0.3$) and the (de)activation was not induced. On the contrary, Supplementary **Figure S1e and f** show that the driving force of the ClC-7 antiporter during the simulations of **Figure 7** in the main text and **Figure S3** respectively, reached values lower than -155 mV. Therefore, a slow (for ClC-7^{WT})/instantaneous (for ClC-7^{fast}) activation was induced. Consequently, we observed small differences in the behaviour of ClC-7^{fast} and ClC-7^{WT}.

3. Steady-state values

Table S3. Steady-state values for simulations shown in Figures 3, 4 and 6.

	Units	Symbol	Figure 3				Figure 4			
			ClC-7[WT]	ClC-7[fast]	ClC-7[unc]	ClC-7[ko]	ClC-7[WT]	ClC-7[fast]	ClC-7[unc]	ClC-7[ko]
Luminal pH		pH_L	4.57	4.57	4.85	4.99	4.57	4.57	4.82	4.92
Luminal chloride concentration	mM	$[Cl^-]_L$	166.2	166.2	29.55	1	160	160	25.98	1
Luminal potassium concentration	mM	$[K^+]_L$	166.7	166.7	49.07	27.63	173.7	173.7	55.82	36.31
Luminal sodium concentration	mM	$[Na^+]_L$	11.49	11.49	3.38	1.9	11.85	11.85	3.85	2.50
Luminal free calcium concentration	mM	$[Ca_f^{2+}]_L$	n.a	n.a	n.a	n.a	0	0	0	0
Total membrane potential	mV	$\Delta\psi_T$	-3.25	-3.25	27.83	42.59	-5.2	-5.2	24.23	34.12

(cont)

	Units	Symbol	Figure 6			
			ClC-7^{WT}	ClC-7^{fast}	ClC-7^{unc}	ClC-7^{ko}
Luminal pH		pH_L	4.58	4.58	4.82	4.92
Luminal chloride concentration	mM	$[Cl^-]_L$	168.1	168.1	26.45	1
Luminal potassium concentration	mM	$[K^+]_L$	165.2	165.2	54.82	35.77
Luminal sodium concentration	mM	$[Na^+]_L$	11.39	11.39	3.78	2.47
Luminal free calcium concentration	mM	$[Ca_f^{2+}]_L$	0.78	0.78	0.09	0.04
Total membrane potential	mV	$\Delta\psi_T$	-3.35	-3.35	24.969	35.96

4. Sensitivity analysis

We investigated the robustness of our model by varying every input parameter by ±10%. The reference scenario was simulated with the initial conditions specified in **Table S1**. Every input parameter of the model listed in **Table S1** was varied in ±10%. Therefore, 66 test scenarios were simulated. For each test scenario we analysed disturbances on the steady-state output values of variables, which are shown in **Table S4**. We calculated the relative difference between the output value obtained from the test simulation (y_{test}) and from the reference simulation (y_{ref}):

$$\text{Relative difference} = \left(\frac{y_{\text{test}} - y_{\text{ref}}}{y_{\text{ref}}}\right) \cdot 100 \quad \text{(S43)}$$

The resulting relative differences are shown in **Figure S8.**

Table S4: Steady-state values of listed variables for reference scenario, simulated with initial conditions specified in **Table S1.**

Description	Units	Symbol	Steady-state value
Luminal pH		pH_L	4.787
Luminal proton concentration	M	$[H^+]_L$	0.049
Luminal chloride concentration	M	$[Cl^-]_L$	0.147
Luminal potassium concentration	M	$[K^+]_L$	0.154
Luminal sodium concentration	M	$[Na^+]_L$	0.011
Luminal total calcium concentration	M	$[Ca_T^{2+}]_L$	0.008
Luminal free calcium concentration	M	$[Ca_f^{2+}]_L$	0.001
Membrane potential	mV	$\Delta\psi$	48.528

5. Supplementary figures

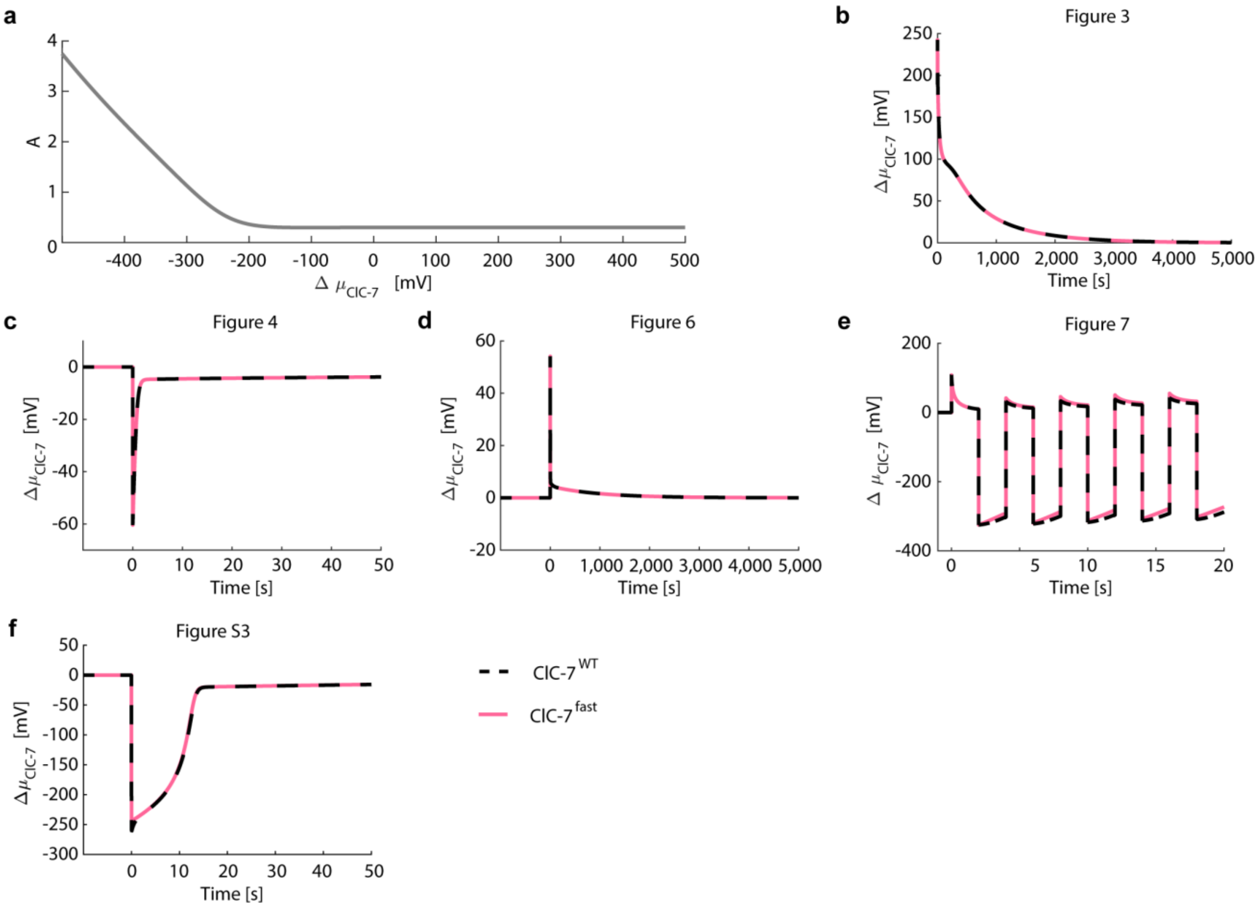

Figure S1. The (de)activation of the ClC-7 antiporter is determined by its driving force. (a) Activity as a function of the driving force. Temporal evolution of the driving force during the simulations shown in **(b)** Figure 3, **(c)** Figure 4, **(d)** Figure 6, **(e)** Figure 7, and **(f)** Figure S3.

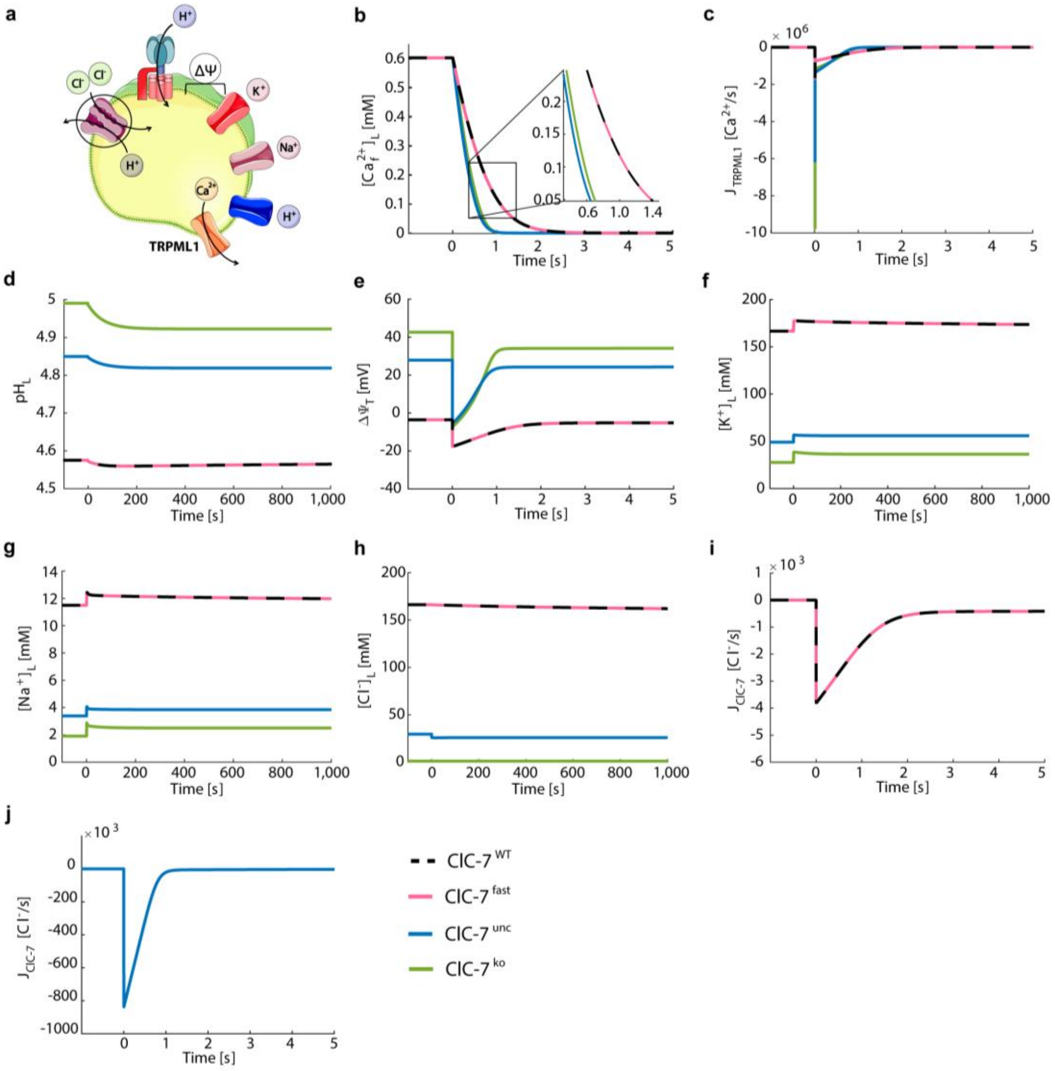

Figure S2. Ca^{2+} release via TRPML1-like channel. (a) Schematic representation of the model with ClC-7 antiporters, V-ATPases, potassium and sodium channels, proton leak, and TRPML1-like channel. The cartoon was created using Servier Medical Art templates (https://smart.servier.com), which are licensed under a Creative Commons License (https://creativecommons.org/licenses/by/3.0/). **(b-j)** Depicted for the different ClC-7 scenarios during triggered calcium release ($ClC\text{-}7^{WT}$, dashed black line; $ClC\text{-}7^{fast}$, red; $ClC\text{-}7^{unc}$, blue; $ClC\text{-}7^{ko}$, green) are luminal free calcium concentration **(b)**, calcium flux via TRPML1-like channel **(c)**, luminal pH **(d)**, total membrane potential **(e)**, luminal concentrations of potassium **(f)**, sodium **(g)** and chloride ions **(h)**, as well as the turnover rates of $ClC\text{-}7^{WT}$ and $ClC\text{-}7^{fast}$ **(i)**, and $ClC\text{-}7^{unc}$**(j)**. The initial conditions were set to the steady-state values of **Figure 3** (**Table S3**).

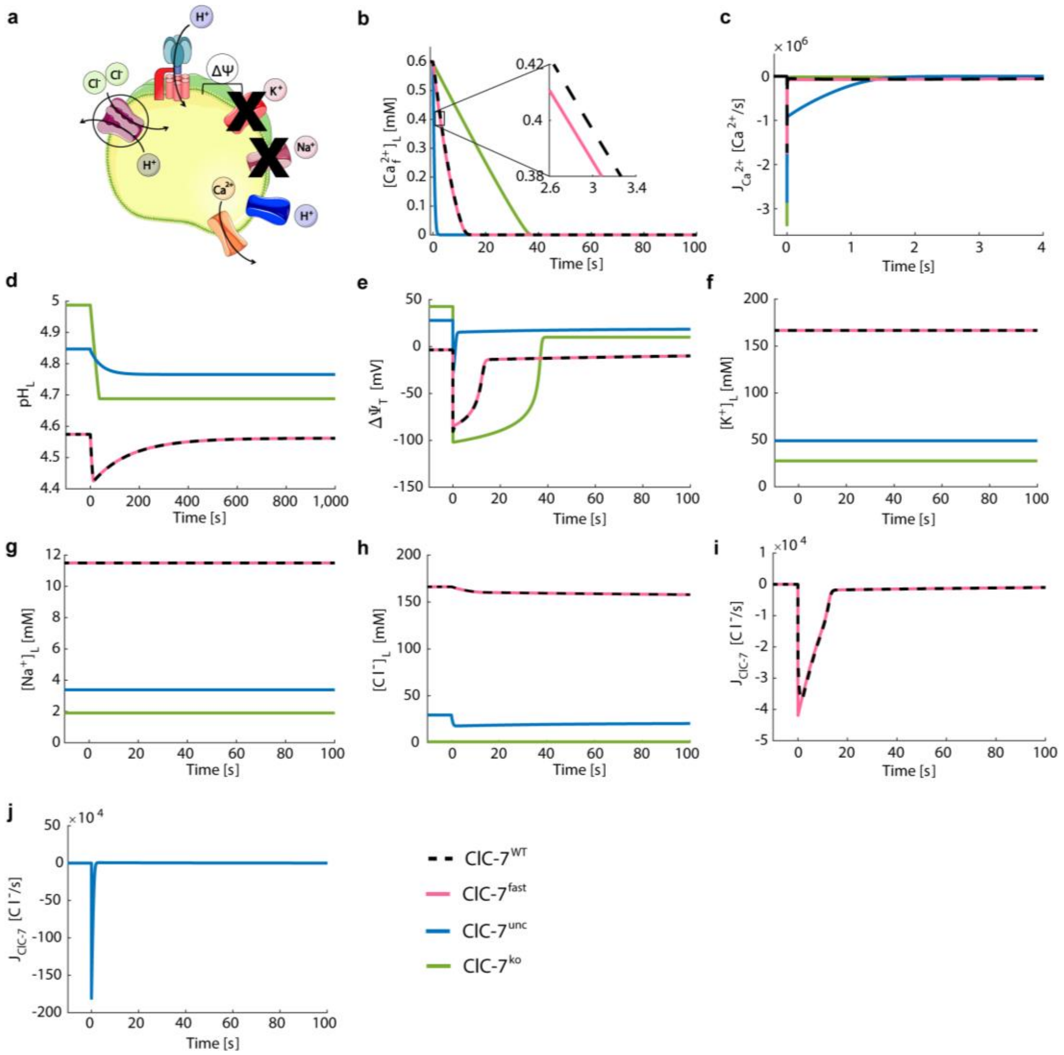

Figure S3. The absence of potassium and sodium conductances highlights the differences in calcium release between ClC-7 scenarios. (a) Schematic representation of the model with ClC-7 antiporters, V-ATPases, proton leak, and calcium release channel. The cartoon was created using Servier Medical Art templates (https://smart.servier.com), which are licensed under a Creative Commons License (https://creativecommons.org/licenses/by/3.0/). **(b-j)** Depicted for the different ClC-7 scenarios during triggered calcium release (ClC-7^{WT}, dashed black line; ClC-7^{fast}, red; ClC-7^{unc}, blue; ClC-7^{ko}, green) are luminal free calcium concentrations **(b)**, calcium flux **(c)**, luminal pH **(d)**, total membrane potential **(e)**, luminal concentrations of potassium **(f)**, sodium **(g)**, and chloride ions **(h)**, as well as the turnover rates of ClC-7^{WT} and ClC-7^{fast} **(i)**, and ClC-7^{unc}**(j)**. The initial conditions were set to the steady-state values of **Figure 3** in the main text (**Table S3**). From t = 0 s, the lysosomal membrane was permeable to calcium ions ($P_{Ca^{2+}} = 8.9 \times 10^{-5}$ cm/s) and impermeable to sodium and potassium ions ($P_{K^+} = P_{Na^+} = 0$).

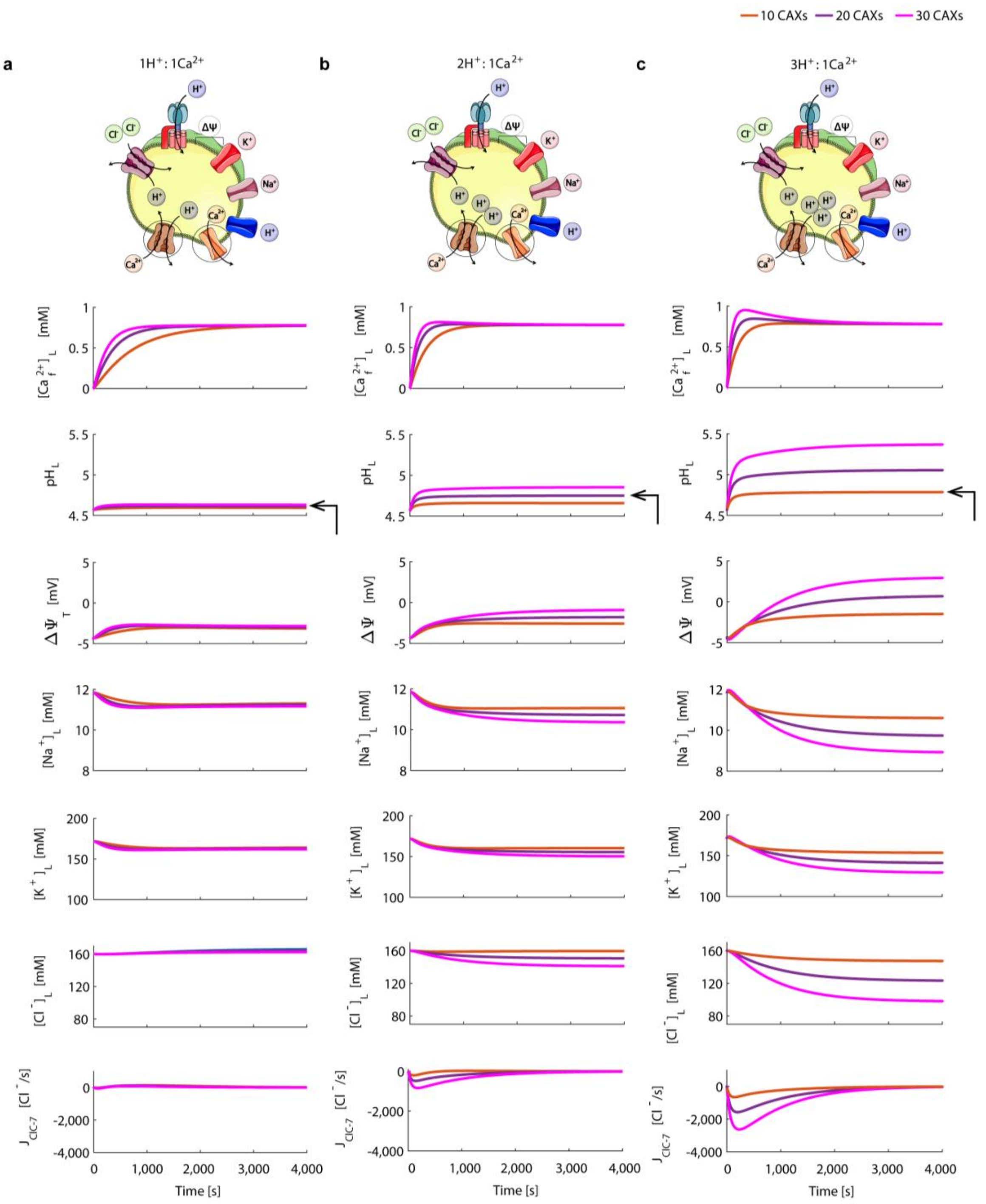

Figure S4. Comparison for different number of CAX and stoichiometries. (a,b,c) Simulations of calcium uptake via CAX in presence of calcium leak for wild-type ClC-7 with different number of CAXs (10,20,30). The cartoons were created using Servier Medical Art templates (https://smart.servier.com), which are licensed under a Creative Commons License (https://creativecommons.org/licenses/by/3.0/). Results are shown for the luminal free calcium concentration, pH, total membrane potential, luminal sodium concentration, luminal potassium concentration and ClC- 7 turnover rate. Simulations were done for three different stoichiometries as depicted **(a)** 1:1, **(b)** 2:1, and **(c)** 3:1. Arrows are indicating the selected cases (based on the steady-state luminal pH) for further analysis: 10 CAX, 3:1 stoichiometry; 20 CAX, 2:1 stoichiometry, and 30 CAX, 1:1 stoichiometry. The initial conditions were set to the steady-state values of **Figure 4** (i.e., after lysosomal calcium release, **Table S3**)

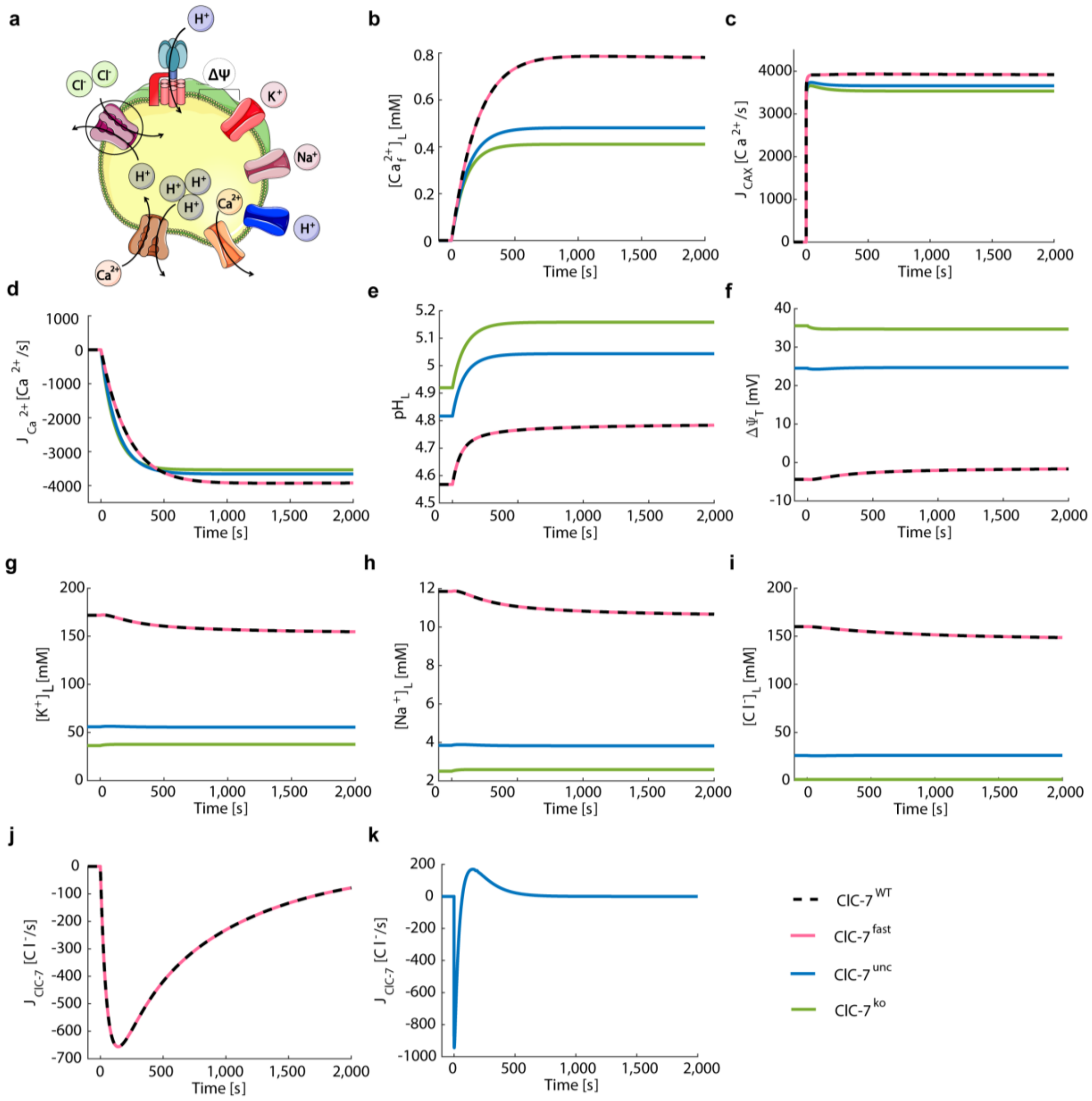

Figure S5. Calcium uptake via 10 CAXs with 3H+:1Ca2+ stoichiometry accompanied by calcium leak. (a) Schematic representation of the model with ClC-7 antiporters, V-ATPases, potassium, sodium channels, proton leak, calcium release channel, and CAXs with $3H^{+}$:$1Ca^{2+}$ stoichiometry. The cartoon was created using Servier Medical Art templates (https://smart.servier.com), licensed under a Creative Commons License (https://creativecommons.org/licenses/by/3.0/). **(b-k)** Depicted for the different ClC-7 scenarios during triggered calcium uptake (ClC-7^{WT}, dashed black line; ClC-7^{fast}, red; ClC-7^{unc}, blue; ClC-7^{ko}, green) are luminal free calcium concentration **(b)**, turnover rate of CAX **(c)**, calcium flux **(d)**, luminal pH **(e)**, total membrane potential **(f)**, luminal concentration of potassium **(g)**, sodium **(h)**, and chloride ions **(i)**, as well as the turnover rates of ClC-7^{WT} and ClC-7^{fast} **(j)**, and ClC-7^{unc} **(k)**. The initial conditions were set to the steady-state values of **Figure 4** (i.e., after lysosomal calcium release, **Table S3**) and the cytosolic calcium concentration was set to 100 nM. From t = 0 s, the lysosomal membrane was permeable to calcium ions ($P_{Ca^{2+}} = 1.5 \times 10^{-7}$ cm/s), and 10 CAX with $3H^{+}$:$1Ca^{2+}$ stoichiometry were turned on.

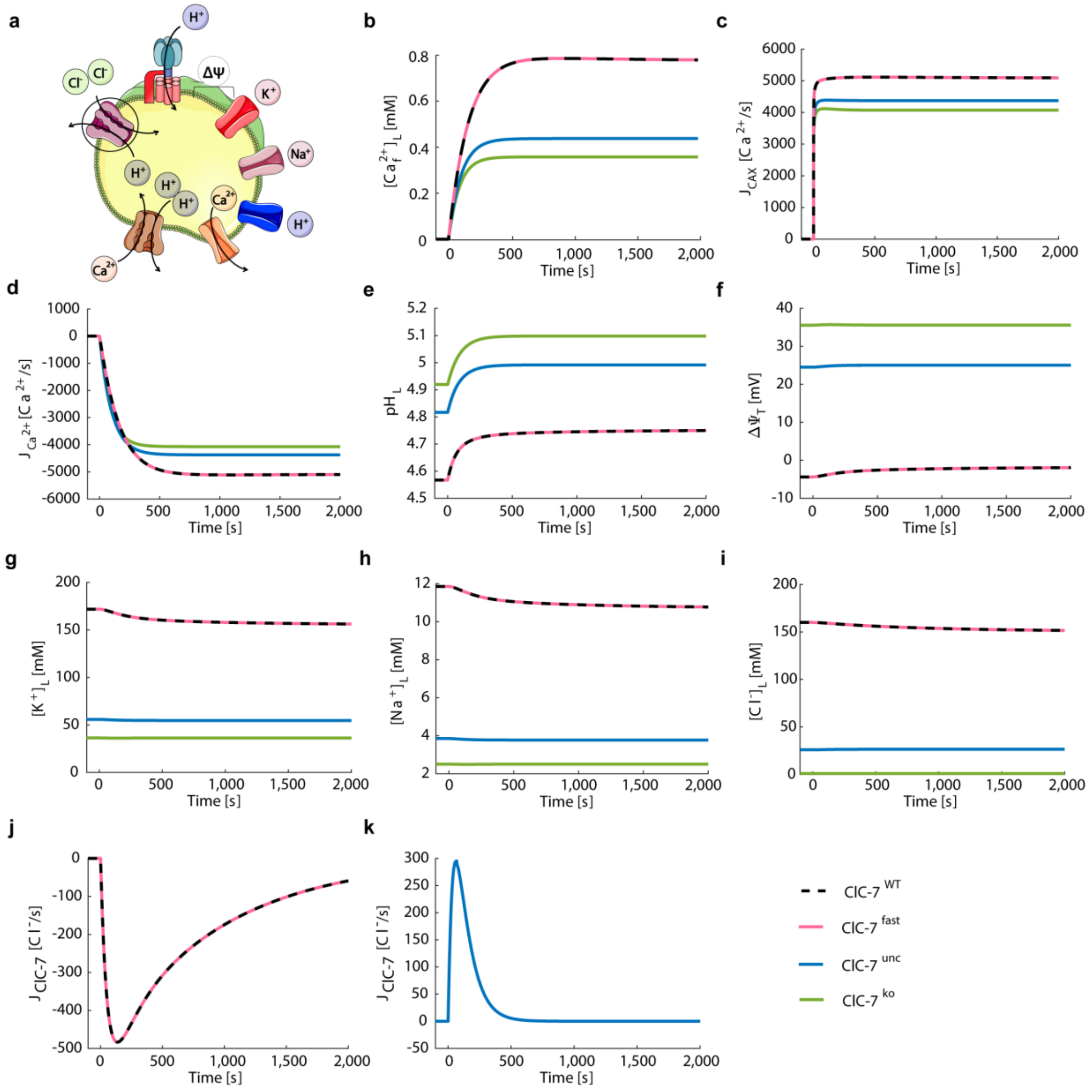

Figure S6. **Calcium uptake via 20 CAXs with 2H+:1Ca2+ stoichiometry accompanied by calcium leak. (a)** Schematic representation of the model with ClC-7 antiporters, V-ATPases, potassium, sodium channels, proton leak, calcium release channel, and CAXs with $2H^{+}$:$1Ca^{2+}$ stoichiometry. The cartoon was created using Servier Medical Art templates (https://smart.servier.com), licensed under a Creative Commons License (https://creativecommons.org/licenses/by/3.0/). **(b-k)** Depicted for the different ClC-7 scenarios during triggered calcium uptake (ClC-7^{WT}, dashed black line; ClC-7^{fast}, red; ClC-7^{unc}, blue; ClC-7^{ko}, green) are luminal free calcium concentration **(b)**, turnover rate of CAX **(c)**, calcium flux **(d)**, luminal pH **(e)**, total membrane potential **(f)**, luminal concentration of potassium **(g)**, sodium **(h)**, and chloride ions **(i)**, as well as the turnover rates of ClC-7^{WT} and ClC-7^{fast} **(j)**, and ClC-7^{unc} **(k)**. The initial conditions were set to the steady-state values of **Figure 4** (i.e., after lysosomal calcium release, **Table S3**) and the cytosolic calcium concentration was set to 100 nM. From t = 0 s, the lysosomal membrane was permeable to calcium ions ($P_{Ca^{2+}} = 2 \times 10^{-7}$ cm/s), and 20 CAX with $2H^{+}$:$1Ca^{2+}$ stoichiometry were turned on.

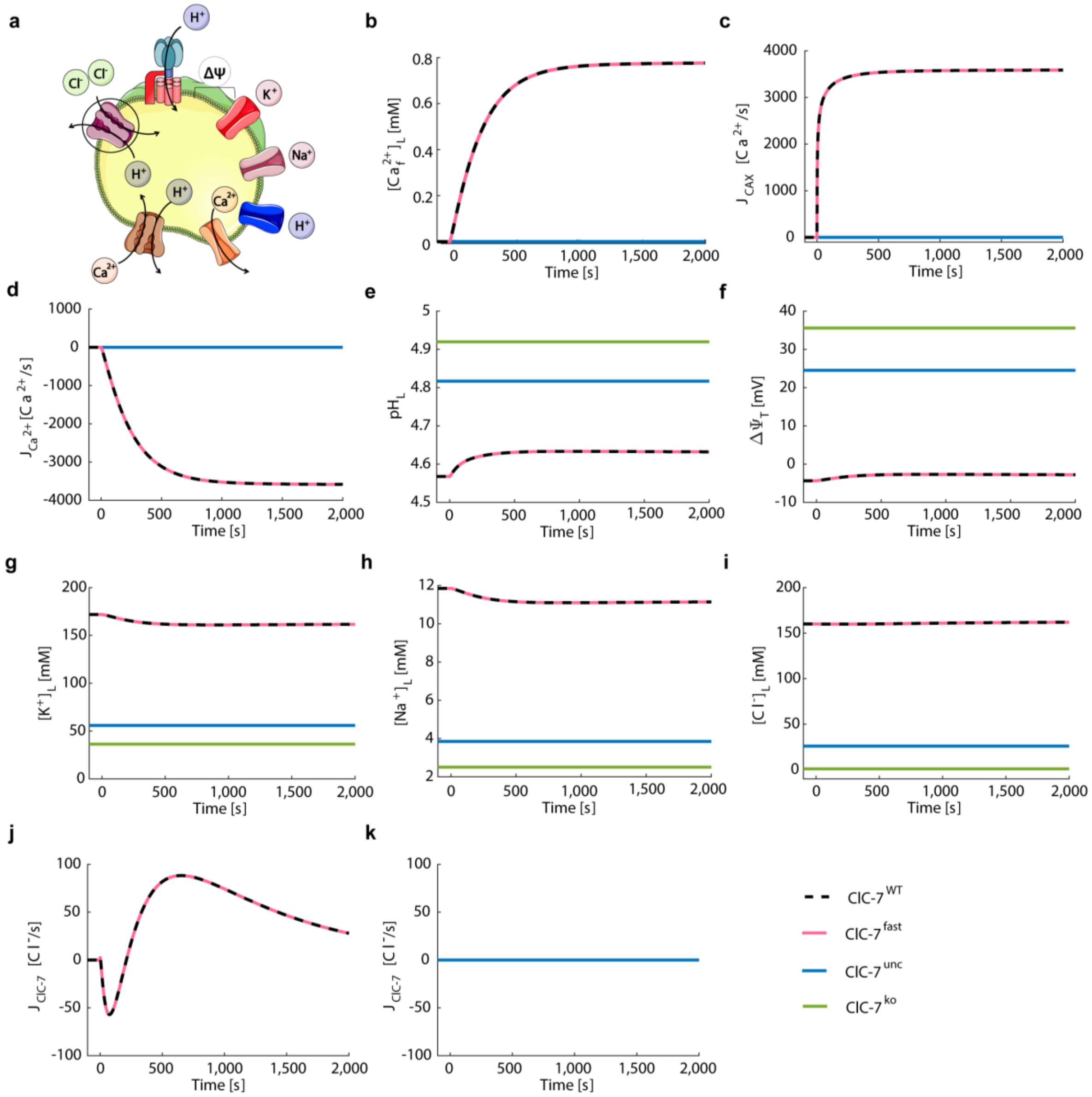

Figure S7. Calcium uptake via 30 CAXs with 1H+:1Ca2+ stoichiometry accompanied by calcium leak. (a) Schematic representation of the model with ClC-7 antiporters, V-ATPases, potassium, sodium channels, proton leak, calcium release channel, and CAXs with $1H^{+}{:}1Ca^{2+}$ stoichiometry. The cartoon was created using Servier Medical Art templates (https://smart.servier.com), licensed under a Creative Commons License (https://creativecommons.org/licenses/by/3.0/). **(b-k)** Depicted for the different ClC-7 scenarios during triggered calcium uptake (ClC-7^{WT}, dashed black line; ClC-7^{fast}, red; ClC-7^{unc}, blue; ClC-7^{ko}, green) are luminal free calcium concentration **(b)**, turnover rate of CAX **(c)**, calcium flux **(d)**, luminal pH **(e)**, total membrane potential **(f)**, luminal concentration of potassium **(g)**, sodium **(h)**, and chloride ions **(i)**, as well as the turnover rates of ClC-7^{WT} and ClC-7^{fast} **(j)**, and ClC-7^{unc} **(k)**. The initial conditions were set to the steady-state values of **Figure 4** (i.e., after lysosomal calcium release, **Table S3**) and the cytosolic calcium concentration was set to 100 nM. From t = 0 s, the lysosomal membrane was permeable to calcium ions ($P_{Ca^{2+}} = 1.4 \times 10^{-7}$ cm/s), and 30 CAX with $1H^{+}{:}1Ca^{2+}$ stoichiometry were turned on.

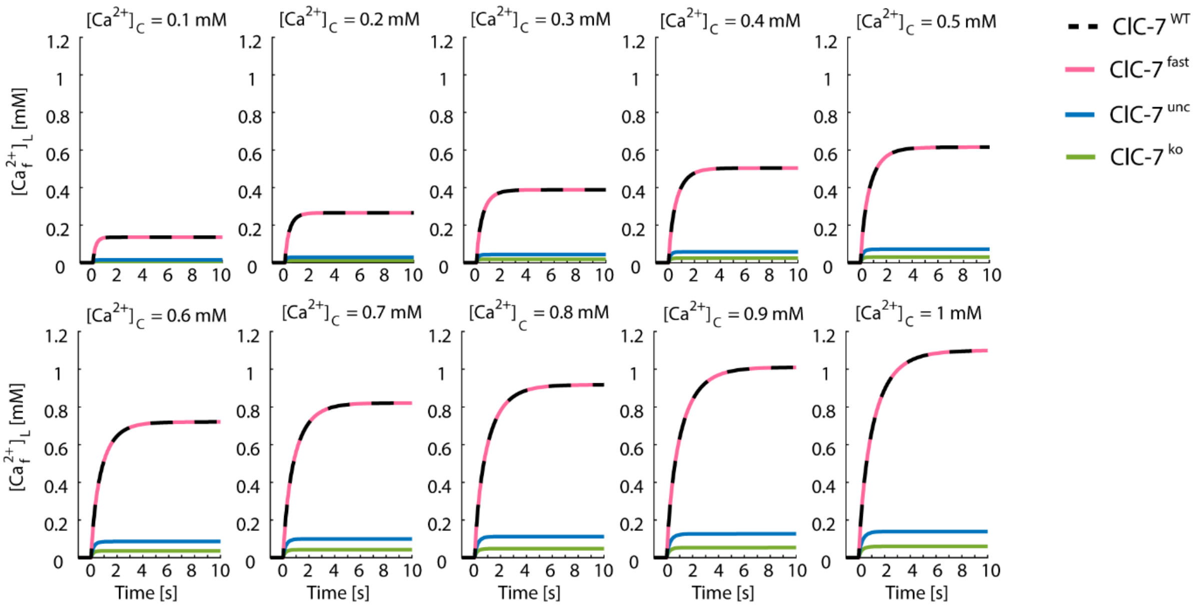

Figure S8. Chloride-proton exchanger supports lysosomal calcium uptake via channel for all the tested values of cytosolic calcium concentration ($[Ca^{2+}]_c$). Luminal free calcium concentration for the different ClC-7 scenarios during calcium uptake (ClC-7^{WT}, dashed black line; ClC-7^{fast}, red; ClC-7^{unc}, blue; ClC-7^{ko}, green) for 10 different values of $[Ca^{2+}]_c$. The initial conditions were set to the steady-state values of **Figure 4** (i.e., after lysosomal calcium release, **Table S3**) and from t = 0 s, the lysosomal membrane was permeable to calcium ions ($P_{Ca^{2+}}$ = 5.7 x 10^{-4} cm/s) representing the opening of the uptake channel.

a

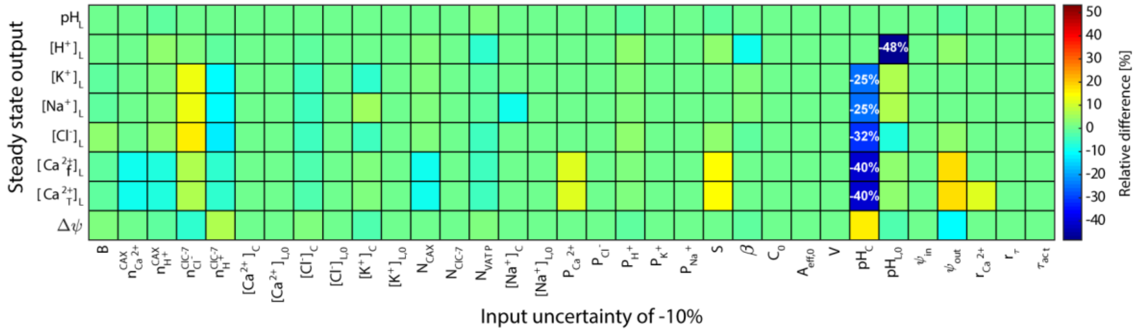

b

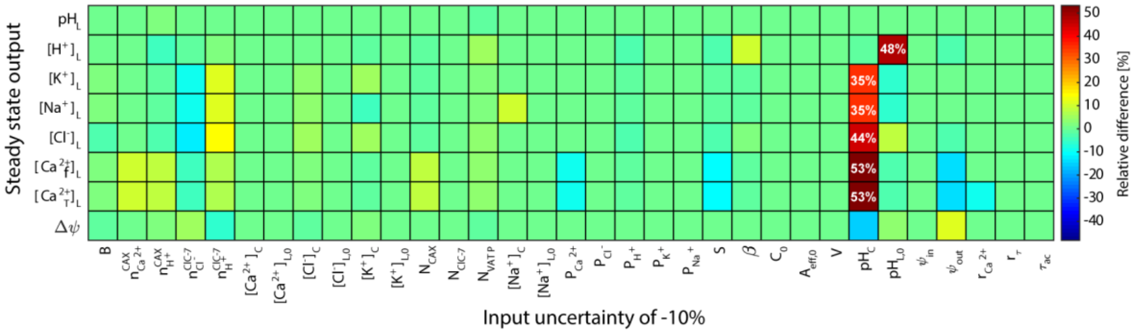

Figure S9. Steady-state luminal pH was robust against changes in model parameters and the steady-state values of luminal ion concentrations were highly affected by variations in cytosolic pH. Each input parameter shown in **Table S1** was varied in **(a)** -10% **(b)** +10%. The colour maps show the relative difference **(Equation S43)** between the steady state value of each variable on the vertical axis under a variation in the input parameter of the horizontal axis and the steady state output for the reference scenario (**Table S4**).

References

1. Rybak, S. L.; Lanni, F.; Murphy, R. F., Theoretical considerations on the role of membrane potential in the regulation of endosomal pH. *Biophys J* **1997,** 73, (2), 674-87.
2. Ishida, Y.; Nayak, S.; Mindell, J. A.; Grabe, M., A model of lysosomal pH regulation. *The Journal of general physiology* **2013,** 141, (6), 705-20.
3. Grabe, M.; Oster, G., Regulation of organelle acidity. *The Journal of general physiology* **2001,** 117, (4), 329-44.
4. Dong, X. P.; Cheng, X.; Mills, E.; Delling, M.; Wang, F.; Kurz, T.; Xu, H., The type IV mucolipidosis-associated protein TRPML1 is an endolysosomal iron release channel. *Nature* **2008,** 455, (7215), 992-6.
5. Xu, H.; Delling, M.; Li, L.; Dong, X.; Clapham, D. E., Activating mutation in a mucolipin transient receptor potential channel leads to melanocyte loss in varitint-waddler mice. *Proc Natl Acad Sci U S A* **2007,** 104, (46), 18321-6.
6. Wu, M. M.; Llopis, J.; Adams, S.; McCaffery, M. J.; Kulomaa, M. S.; Machen, T. E.; Moore, H.-P. H.; Tsien, R. Y., Organelle pH studies using targeted avidin and fluorescein-biotin. *Chemistry & Biology* **2000,** 7, (3), 197-209.
7. Roos, A.; Boron, W. F., Intracellular pH. *Physiol Rev* **1981,** 61, (2), 296-434.
8. Alberts, B.; Wilson, J. H.; Hunt, T., *Molecular biology of the cell*. 5th ed.; Garland Science: New York, 2008; p xxxiii, 1601, 90 p.
9. Clapham, D. E., Calcium signaling. *Cell* **2007,** 131, (6), 1047-58.
10. Steinberg, B. E.; Huynh, K. K.; Brodovitch, A.; Jabs, S.; Stauber, T.; Jentsch, T. J.; Grinstein, S., A cation counterflux supports lysosomal acidification. *J Cell Biol* **2010,** 189, (7), 1171-86.
11. Christensen, K. A.; Myers, J. T.; Swanson, J. A., pH-dependent regulation of lysosomal calcium in macrophages. *J Cell Sci* **2002,** 115, (Pt 3), 599-607.
12. Hartmann, T.; Verkman, A. S., Model of ion transport regulation in chloride-secreting airway epithelial cells. Integrated description of electrical, chemical, and fluorescence measurements. *Biophys J* **1990,** 58, (2), 391-401.
13. Leisle, L.; Ludwig, C. F.; Wagner, F. A.; Jentsch, T. J.; Stauber, T., ClC-7 is a slowly voltage-gated $2Cl^-/1H^+$-exchanger and requires Ostm1 for transport activity. *EMBO J* **2011,** 30, (11), 2140-52.
14. Blackford, S.; Rea, P. A.; Sanders, D., Voltage sensitivity of H+/Ca2+ antiport in higher plant tonoplast suggests a role in vacuolar calcium accumulation. *J Biol Chem* **1990,** 265, (17), 9617-20.
15. Ludwig, C. F.; Ullrich, F.; Leisle, L.; Stauber, T.; Jentsch, T. J., Common gating of both CLC transporter subunits underlies voltage-dependent activation of the 2Cl-/1H+ exchanger ClC-7/Ostm1. *J Biol Chem* **2013,** 288, (40), 28611-9.
16. Hille, B., *Ion channels of excitable membranes*. Third edition ed.; p xviii, 814 pages.
17. Samtleben, S.; Jaepel, J.; Fecher, C.; Andreska, T.; Rehberg, M.; Blum, R., Direct imaging of ER calcium with targeted-esterase induced dye loading (TED). *J Vis Exp* **2013**, (75), e50317.
